# Evidence-Based Effects of High-Intensity Interval Training on Exercise Capacity and Health: A Review with Historical Perspective

**DOI:** 10.3390/ijerph18137201

**Published:** 2021-07-05

**Authors:** Muhammed Mustafa Atakan, Yanchun Li, Şükran Nazan Koşar, Hüseyin Hüsrev Turnagöl, Xu Yan

**Affiliations:** 1 Division of Exercise Nutrition and Metabolism, Faculty of Sport Sciences, Hacettepe University, 06800 Ankara, Turkey; muhammed.atakan@hacettepe.edu.tr (M.M.A.); nazank@hacettepe.edu.tr (Ş.N.K.); deniz@hacettepe.edu.tr (H.H.T.); 2 China Institute of Sport and Health Science, Beijing Sport University, Beijing 100192, China; 3 Institute for Health and Sport (iHeS), Victoria University, Melbourne 8001, Australia; xu.yan@vu.edu.au; 4 Sarcopenia Research Program, Australia Institute for Musculoskeletal Sciences (AIMSS), Melbourne 3021, Australia

**Keywords:** exercise, intermittent training, physical endurance, health benefits, physiological adaptation

## Abstract

Engaging in regular exercise results in a range of physiological adaptations offering benefits for exercise capacity and health, independent of age, gender or the presence of chronic diseases. Accumulating evidence shows that lack of time is a major impediment to exercise, causing physical inactivity worldwide. This issue has resulted in momentum for interval training models known to elicit higher enjoyment and induce adaptations similar to or greater than moderate-intensity continuous training, despite a lower total exercise volume. Although there is no universal definition, high-intensity interval exercise is characterized by repeated short bursts of intense activity, performed with a “near maximal” or “all-out” effort corresponding to ≥90% of maximal oxygen uptake or >75% of maximal power, with periods of rest or low-intensity exercise. Research has indicated that high-intensity interval training induces numerous physiological adaptations that improve exercise capacity (maximal oxygen uptake, aerobic endurance, anaerobic capacity etc.) and metabolic health in both clinical and healthy (athletes, active and inactive individuals without any apparent disease or disorder) populations. In this paper, a brief history of high-intensity interval training is presented, based on the novel findings of some selected studies on exercise capacity and health, starting from the early 1920s to date. Further, an overview of the mechanisms underlying the physiological adaptations in response to high-intensity interval training is provided.

## 1. Introduction

Exercise is a cornerstone in the primary prevention of chronic diseases including diabetes mellitus, cancer, obesity, hypertension, coronary heart disease, cardiovascular disease, and depression [1,2,3]. For centuries, exercise has long been prescribed by physicians as a medicine for their patients [4], and quotes attributed to Hippocrates, the father of Western medicine, include: “Walking is man’s best medicine” and “if there is a deficiency in food and exercise, the body will fall sick [5,6].” Evidence-based scientific guidelines suggest that exercise is a novel non-pharmacological strategy in the prevention and management of chronic diseases [7,8,9,10,11]. Despite these well-documented benefits of exercise, a third of adults and four-fifths of adolescents—approximately 1.4 billion people—do not meet public health guidelines for recommended levels of exercise [7], making physical inactivity a global problem [12]. The recently updated World Health Organization 2020 Guidelines on physical activity and sedentary behavior [13] recommend performing at least 150 to 300 min of moderate-intensity physical activity, or 75 to 150 min of vigorous-intensity aerobic exercise, per week to achieve substantial health benefits [14]. However, the lack of sufficient time is the most common barrier to adhering to regular exercise [15]. Therefore, research has recently focused on interval training models, which consist of brief periods of intense activity performed with a “near-maximal” or “all-out” effort corresponding to ≥90 of maximal oxygen uptake ($\dot{V}$O_2max_) [16] or >75% of maximal power [17], interspersed with passive or active recovery periods, that can induce similar or even greater physiological adaptations when compared to moderate-intensity continuous training (MICT) [17,18,19], which generally consists of 30–60 min of moderate-intensity exercise at 40% to <60% of oxygen consumption reserve [20,21]. There are numerous excellent reviews on the effects of interval training on exercise capacity and general health in healthy and clinic populations as well as the mechanisms underlying these effects [15,18,22,23,24]. However, to the best of our knowledge, no study to date has applied a historical approach. In this review, after defining the types of different interval-training models, the findings of some selected high-intensity interval-training studies that have received considerable interest due to their novel findings on exercise capacity and health, which can be considered milestone studies in the related literature, are presented. Finally, a brief overview of the mechanisms underlying the physiological adaptations in response to high-intensity interval training is presented.

## 2. Interval Training

Interval training is characterized by short bursts of intense activities that elicit ≥90% of $\dot{V}$O_2max_ [16], >75% of maximal power [17] or supra-maximal effort [16,17,18], with periods of rest or low-intensity exercise for recovery. The most used types of interval training models are: (1) the high-intensity interval training (HIIT) model with submaximal efforts that elicit ≥90% of $\dot{V}$O_2max_ [16] or >75% of maximal power [18,25]; (2) the sprint interval training (SIT) model, a more intense version of HIIT that involves maximal or supramaximal efforts greater than $\dot{V}$O_2max_ or maximal power; and (3) the repeated-sprint training (RST) model, which is characterized by performing a high number of sprints lasting less than 10 s interspersed with relatively shorter recoveries (<60 s) compared to the recovery periods of SIT [26].

Interval training has been an integral part of training for coaches and athletes to enhance performance for over a century, and it has received considerable scientific inquiry due to its ability to induce remarkable physiological adaptations and health benefits that resemble MICT with less time commitment [27]. Besides, it should be noted that from a mechanistic standpoint, physiological adaptations elicited by interval training is not only attributable to intensity per se but also the intrinsic nature of the intermittent exercise.

It is well documented that HIIT provides a robust stimulus for central cardiovascular adaptations and metabolic stress [28], while MICT mainly triggers peripheral adaptations contributing to muscular oxygen extraction and metabolic efficiency [29,30,31]. HIIT is known to elicit higher enjoyment than MICT, as reported by original research [32] and meta-analysis [33], making the interval training model a practical and enjoyable exercise mode for the general population. Furthermore, a meta-analysis by Reljic et al. [34] reported that following HIIT-based interventions, there were lower dropout rates than traditional exercise programs in previously sedentary individuals, showing that HIIT is tolerable and acceptable. However, a single bout of high-intensity interval exercise performed at a strenuous intensity with a low resting period likely results in decreased enjoyment, and thus, adequate resting intervals between high-intensity sessions are essential in preventing negative affective responses for long-term exercise maintenance.

**Figure 1:**
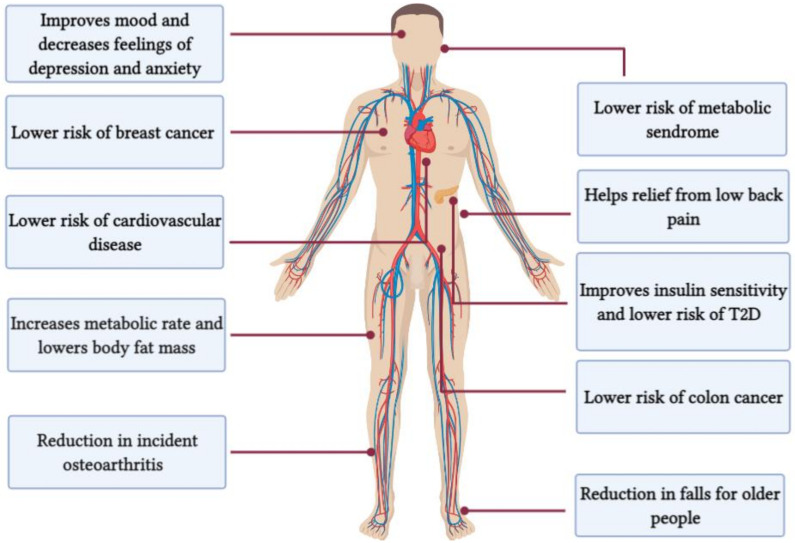
Documented health benefits of high-intensity interval training.

Due to its time-saving nature and the induced physiological adaptations similar to MICT, HIIT has been ranked first in 2018 [35] and third in 2019 in Worldwide Fitness Trends [36]. Growing evidence-based research, both original research and meta-analysis studies, shows that interval training programs ranging from 5 days to 12 months are effective in improving $\dot{V}$O_2max_ [37], endurance capacity [38,39], resting metabolic rate [40], substrate metabolism [41,42], body composition [43], insulin sensitivity [44,45], and cognitive functions [46,47,48]. Besides, interval training has been shown to decrease the risk for cardiovascular diseases [15,25], breast cancer [49], metabolic syndrome [50], osteoarthritis [51,52], and rheumatoid arthritis known to cause lower back pain [53,54,55,56,57] (Figure 1). In the following subheadings, the definition, as well as health and performance benefits, of different interval trainings models are presented.

### 2.1. High-Intensity Interval Training

High-intensity interval exercise is characterized by relatively short bursts of vigorous activity performed at a high relative workload corresponding to ≥90% of $\dot{V}$O_2max_ [16], >75% of maximal power [17], ≥90% minimal running speed required to elicit VO_2max_ [16], and at a range of “hard” to “very hard” rate of perceived exertion (≥6 on a 10 Borg scale and ≥15 on a 6–20 scale) [16]. Each single effort generally lasts from a few seconds to several minutes, dependent on exercise intensity, with multiple efforts interspersed by up to a few minutes of rest or less exertion. Although some research has determined the exercise intensity of high-intensity work based on the HR in the absence of equipment and expertise for determining $\dot{V}$O_2max_ [21,58], it is not consistently reliable due to the heterogeneous character of the heart rate (HR), such as people with cardiovascular disease, who can have an upward deflection of the HR performance curve [59]; using maximal HR (HR_max_) for exercise intensity can result in an overestimation of the individual training HR up to 40% in single cases [60,61], and true HR_max_ can not be obtained in untrained subjects using incremental exercise tests on a treadmill or a cycle ergometer, because of local leg fatigue that is likely to cause tests to end prematurely before cardiopulmonary endpoints [62].

Despite having various sub-categories, HIIT is generally sub-categorized into low—less than 15 min—and high-volume (greater than 15 min) HIIT based on the total time spent in active intervals [63]. These HIIT protocols have been shown to lead to similar, and sometimes greater, health and performance benefits despite less time commitment [43,64,65,66,67]. Moreover, as highlighted by a recent review [63] and meta-analysis [43], it is evident that low-volume HIIT, involving less than 15 min of active high-intensity training per session, is a time-efficient exercise strategy to improve cardiometabolic health and cardiovascular endurance [63].

Inevitably, strong motivation is required due to the high-exertion nature of the classic interval training model, which is very fatiguing and too strenuous for sedentary individuals [19]. In addition, the total time commitment of classic HIIT protocols include warm-up, recovery intervals, and cool-down, which is typically more than 20 min and therefore reduces the time efficiency [64]. In this sense, the findings of studies involving low-volume protocols are promising to determine whether the levels of exertion and time-commitment of the current HIIT protocols can be reduced while maintaining the associated health and performance benefits.

### 2.2. Sprint Interval Training

SIT is a more intense form of HIIT performed at an intensity that exceeds the workload required to elicit $\dot{V}$O_2max_ or maximal power. Each bout of high-intensity work lasts short periods of activity (≤30 s), separated by relatively long recovery periods between intervals (~4 min) [68]. The target exercise intensities during SIT are usually workloads greater than the pace that elicits $\dot{V}$O_2max_ or maximal power, including ‘all-out’ or ‘supramaximal’ efforts [68]. The minimal intensities suggested for SIT are >100% of maximal power [17], ≥85% maximal sprinting speed, and ≥160% minimal running speed required to elicit VO_2max_ [16]. The most used SIT protocol is performed on a cycle ergometer and consists of 4 × 30 s all-out maximal intervals, pedaling against a high force corresponding to approximately 170% of $\dot{V}$O_2max_ with rest intervals or light exercise [69].

Ample evidence has shown that 2 and 6 weeks of SIT was effective in increasing skeletal muscle oxidative capacity and endurance capacity as well as inducing key molecules associated with mitochondrial biogenesis in healthy individuals [70,71,72], which was comparable to that observed after MICT, despite robust difference in training volume. Besides, some research has reported improved insulin sensitivity in young healthy men [45,73] and increased resting fat oxidation in overweight/obese individuals following 2 weeks [41], as well as reduced fat mass following 12 and 15 weeks of SIT in normal weight and overweight individuals [74,75]. However, it is worth noting that SIT requires maximal effort and may not be well-tolerated or appealing for many individuals, including people with chronic diseases or obesity in particular. Hence, although current findings seem interesting from the perspective of health and performance benefits, it would not be an easy translation into physical activity recommendations for the general population, suggesting the need for further research into SIT that can establish acceptable and effective protocols.

### 2.3. Repeated-Sprint Training

RST is characterized by performing repeated sprints (10–20 maximal or shuttle sprints lasting ≤10 s), interspersed with short recoveries (<60 s) [76,77]. The minimal intensity suggested for RST is ≥120% minimal running speed required to elicit VO_2max_ [16]. This training model is widely utilized in the physical preparation of athletes for many sports [78,79,80]. The published literature is not, however, as abundant for RST studies as for SIT or HIIT. The SIT and HIIT models are repeatedly performed with recovery periods long enough to allow for near full recovery of sprint performance, whilst the recovery duration between repeated sprints during RST is minimal, thus there is inevitably performance decrement during RST. Research has shown that RST can improve endurance, sprint, repeated-sprint ability, and aerobic capacity in healthy and fit subjects [26,77]. A meta-analysis by Taylor et al. [81] reviewed controlled and non-controlled trials investigating the effect of RST on athletic-performance variables, including counter-movement jump, 10 m sprint, 20 m sprint, 30 m sprint, repeated-sprint ability, and high-intensity intermittent-running performance, and documented RST to be effective in improving power, speed, repeated-sprint ability, and endurance.

## 3. Effects of HIIT on Exercise Capacity and Health Based on Selected Studies

**Figure 2:**
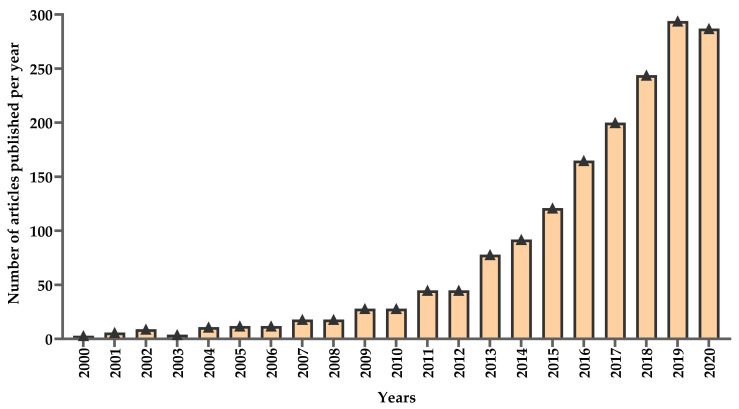
The number of high-intensity interval-training articles published in 2000–2020.

In this section, early attempts applying interval training and the main findings of selected HIIT studies are summarized. Since the early 2000s, the number of HIIT studies has gained momentum and increased exponentially. A quick search on PubMed now yields about 300 research articles per year containing “high-intensity interval training”, “sprint interval training”, “repeated sprint training” and “HIIT” in the title (Figure 2). Some HIIT studies to date have significantly contributed to the literature, by showing the physiological and molecular adaptations in response to high-intensity interval exercise intervention in healthy and clinical populations. The training studies included in this section are described in Table 1.

### 3.1. Most Early Attempts at Applying Interval Training Model

Despite the momentum in the number of studies indicating the effectiveness of HIIT in improving health and exercise performance over the last 20 years, it is well known that this training model dates back a hundred years and was the key strategy behind important sporting successes observed in the early 20th century [27]. During this early period, HIIT significantly evolved through the contributions of innovative athletes and trainers [27,51], such as Lauri Pikhala, who coached champion runners in Finland, including Hannes Kolehmainen and Paavo Nurmi. Paavo Nurmi was the world’s most successful distance runner between 1920 and 1930, earning his place in sporting history with nine Olympic gold medals; his training program mainly consisted of 20 × 60 s of high-intensity intervals, separated by short rest periods.

In the 1930s, a German physician and coach Woldemar Gerschler, together with cardiologist Herbert Reindel, designed a training model performed at a specific HR with rest periods. In this model, the athletes performed a short-distance run with a target HR of 180 beats/min, followed by a rest period until their HR decreased to 120 beats/min, before starting the next repetition [102]. As reviewed by Gibala and Hawley [102], Gerschler and Reindel proposed that the rest period between high-intensity bouts was the most important aspect of this training model, during which the heart adapted, allowing it to grow larger and stronger. This notion was supported by a study they conducted, in which 21 days of HIIT elicited a 20% increase in heart volume and improvement in the endurance capacity of middle- and long-distance runners [102]. Perhaps the most notable athletes in history to adopt the interval training model is Sir Roger Bannister, who first ran 1.6 km (1 mile) in less than 4 min. His training protocol involved 10 × 400 m of running under 60 s, separated by 2 min rest [102].

### 3.2. Studies on Exercise Capacity and Health in Healthy Populations

Although trainers and athletes have long known the effectiveness of interval training since the early 20th century, the effects of interval training models on human physiology did not get enough attention until the early 1960s [103]. In the following years, the effects of this type of exercise model on health and exercise performance gained popularity [82,83,95,104]. For example, in 1973, the Bengt Saltin group reported approximately 20% increase in $\dot{V}$O_2max_ in young military conscripts following only 2 months of an interval training program consisting of 15 s exercise, followed by 15 s rest or 3 min exercise, followed by 3 min rest (3 times/wk) [82]. In 1975, Fox et al. reported [83] that the improvement in $\dot{V}$O_2max_ in young healthy males was dependent on training intensity and attributed to increased stroke volume (SV) and arteriovenous oxygen difference (a-vO_2_ diff) following 7 and 13 weeks of interval training program performed on an indoor oval track. In 1976, Henriksson and Reitman aimed to reveal if the effects of HIIT with MICT on oxidative and glycolytic enzyme activities were muscle-fiber-specific [84]. To address this, nine young healthy subjects were divided into HIIT (5 × 4 min cycling at 101% $\dot{V}$O_2max_, separated by 2 min rest) or MICT (27 min of continuous cycling at 79% $\dot{V}$O_2max_) groups for 7–8 weeks [84]. The authors reported an increase in $\dot{V}$O_2max_ and the type II-succinate-dehydrogenase activity only in the HIIT group, while the MICT group showed an average 32% increase in type I-succinate-dehydrogenase activity [84]. This paper showed that the enhancement of the $\dot{V}$O_2max_ and the physiological adaptations observed in skeletal-muscle-fiber types following exercise were dependent on exercise intensity and fiber recruitment during exercise. Following these studies, Roberts et al. investigated in early 1980 the effects of HIIT on the anaerobic metabolism of skeletal muscle in young active males [85]. The training program consisted of eight 200 m runs at 90% of maximal speed with 2 min rest periods over 5 weeks [85]. They reported an improved anaerobic metabolism, as evidenced by the increased activities of key enzymes associated with glycogenolysis and anaerobic glycolysis in skeletal muscle [85], indicating that a short-term interval training program was a potent stimulus to improve anaerobic metabolism.

In 1986, Sharp et al. documented that 8 weeks of SIT involving 8 × 30 s all-out training (4 times/week) increased the buffering capacity and $\dot{V}$O_2max_ by 37% and 8%, respectively, in young healthy men [86]. This paper was the first to show SIT increased muscle buffer capacity [86]. Furthermore, in 1996 Tabata et al. [87] compared the effects of 6 weeks of HIIT (8 × 20 s at 170% PPO, separated by 10 s rest, 5 days/week) and MICT (70% $\dot{V}$O_2max_, 60 min/day, 5 days/week) on both aerobic and anaerobic exercise capacity in active individuals. This novel research showed that despite the robust difference in time commitment between the exercise protocols administered (43 min/week versus 300 min/week), HIIT was more effective in simultaneously upregulating oxidative and glycolytic energy systems, in turn producing an improved energy state in the exercising muscle and preserving high-energy phosphates [87].

Following this study, interest in the potential value of different interval training models that could be applied as an alternative to traditional MICT gained momentum. For example, MacDougall et al. in 1998 reported an increase in $\dot{V}$O_2max_ and glycolytic and oxidative enzyme activity, including citrate synthase (CS), succinate-dehydrogenase, and malate dehydrogenase, following 7 weeks of SIT involving 4 to 10 × 30 s all-out with 2 to 4 min of recovery (3 times/week) in young healthy men [89]. In 2006, a landmark study by Gibala et al. was published, which compared SIT with traditional MICT on exercise performance and skeletal-muscle adaptations [71]. Despite completing ~90% less total training volume (630 kJ vs. 6500 kJ), six sessions of SIT (30 s × 4–6 repeats) resulted in similar improvement in time trial performance to that observed following MICT [71]. Analyses of skeletal-muscle samples showed similar increases in muscle oxidative capacity, muscle buffering capacity, and glycogen content between the two groups [71]. The authors, therefore, concluded that SIT was a time-efficient strategy for rapid adaptations in exercise performance and skeletal muscle [71], showing SIT to be as effective as MICT in improving cardiorespiratory fitness, muscle buffering capacity, and glycogen content, despite less time commitment.

Notably, in 2007, Helgerud et al. compared four training methods in young healthy males (2 interval training versus 2 continuous training) performed at different intensities, matched for total work and frequency (3 days/week for 8 weeks) [90]. The interval groups performed either 15/15 interval running (15 s of running at 90–95% HR_max_ followed by 15 s of active resting at 70% HR_max_) or 4 × 4 min of interval running at 90–95% HR_max_ followed by 3 min of active resting at 70% HR_max_. The continuous training involved either slow long-distance running at 70% HR_max_ or lactate threshold running at 85% HR_max_. The authors reported an increase in $\dot{V}$O_2max_ and left ventricular SV in the two HIIT groups, but no changes in the continuous running groups [90]. The authors, therefore, concluded that HIIT was more effective in improving $\dot{V}$O_2max_ and SV than performing the same total work at either lactate threshold or at 70% HR_max_ [90]. This paper revealed for the first time that increases in $\dot{V}$O_2max_ in response to interval training corresponded with changes in SV, showing a close link between the two.

On the other hand, until a study by Little et al. [91], the majority of HIIT studies applied “all out” protocol that is not practical and is poorly tolerated by certain individuals; thus, there was a necessity to design a more practical and attainable protocol capable of inducing similar adaptations as classic HIIT models for the general population. To address this, in 2010, Little et al. [91] designed a practical model of low-volume high-intensity interval exercise (8 to 12 × 60 s intervals at ~100% of PPO, separated by 75 s of recovery) [91]. Similar to previous studies, they reported that performing this new training model over 2 weeks increased exercise capacity, as shown by improvements in cycling time trial performance [91]. Additionally, the protein content and maximal activity of CS and cytochrome C oxidase in skeletal muscle increased, together with the protein content of mitochondrial transcription factor A and sirtuin 1, as well as the nuclear abundance of peroxisome proliferator-activated receptor gamma coactivator 1 alpha (PGC-1α) [91], which mediated skeletal-muscle mitochondrial adaptations. This is the first study to document that this new exercise model is a potent stimulus for improving endurance performance, skeletal muscle mitochondrial capacity, sirtuin 1, mitochondrial transcription factor A, and nuclear PGC-1α, which were previously reported to increase in response to the repeated SIT protocols of earlier studies [76,77,99,100].

The following years have witnessed some novel research involving mechanistic examinations carried out with muscle biopsies. These studies have allowed researchers to both investigate the effects of different types of interval training models on mitochondrial respiration and mitochondrial biogenesis as well as distinguish the effects of training volume and training intensity. For example, in 2016, one of these several HIIT studies by Granata et al. compared three different training methods on skeletal-muscle mitochondrial content and mitochondrial respiration: SIT (4–10 × 30 s all-out bouts at ~200% of PPO), HIIT (4–7 × 4 min intervals at ~90% PPO), or sub-lactate threshold continuous training (20–36 min at ~65% PPO)), performed 3 sessions/wk for 4 weeks [92]. Unlike previously published data, PPO improved in the HIIT and SIT groups, while the time trial performance only improved in sub-lactate threshold continuous training and HIIT groups, remaining unchanged after SIT [92]. Only SIT increased mass-specific mitochondrial respiration in skeletal muscle, as well as the protein content of PGC-1α, protein p53, and plant homeodomain finger-containing protein 20 [92], which modulate mitochondrial biogenesis [105]. The authors concluded that training intensity was an important factor that determines changes in mitochondrial respiration, suggesting that SIT promotes greater and faster mitochondrial adaptations in skeletal muscle. Further, an elegant follow-up study by the same group was conducted to ascertain how different training volumes would affect mitochondrial respiration and markers of mitochondrial biogenesis [93]. To address this, ten healthy men performed high-intensity interval cycling during 3 consecutive training phases, 4 weeks of normal-volume training (3 days/week), followed by 20 days of high-volume training (2 sessions/day) and 2 weeks of reduced-volume training (5 sessions) [93]. The main finding of the study was that mitochondrial parameters did not change following normal-volume training, while there were improvements in mitochondrial respiration, the maximal activity of CS, and PGC-1α after high-volume training [93]. Similarly, the protein content of mitochondrial complex I, II, III, IV, and V, and mass-specific mitochondrial respiration were elevated after high-volume training but dropped quickly after 2 weeks of reduced-volume training [93]. The authors concluded that there would be a rapid reversal of human skeletal-muscle adaptations to a reduction in training volume, and that training volume plays a key role in training-induced mitochondrial adaptations.

Finally, in the growing HIIT literature, most studies examining the effects of HIIT on health and performance involved high-intensity interval exercise performed once a day for various durations [106,107,108,109,110,111,112,113,114]. This has led Andrade-Souza and colleagues to wonder whether the effects of “twice a day” and “once a day” HIIT on mitochondrial biogenesis were identical. To address this, this research group designed a novel protocol that allowed the authors to manipulate the recovery duration between the first muscle glycogen-depleting exercise and the second exercise session, either 2 h (twice-a-day) or 15 h (once-daily) after the first exercise session [115]. In both approaches, the second exercise session (10 × 2 min intervals corresponding to an intensity of 20% of the difference between the lactate threshold and PPO) started with reduced muscle glycogen content [115]. The authors reported greater mRNA expression of PGC-1α, peroxisome proliferator-activated receptor, and peroxisome proliferator-activated receptor delta, and greater nuclear abundance of PGC-1α and protein p53 after twice-a-day high-intensity interval exercise [115]. On the other hand, muscle glycogen decreased similarly between the two exercise approaches [115]. A notable finding in this study is that performing two exercise sessions separated by a short recovery period might be more effective at inducing adaptations related to mitochondrial biogenesis and fat oxidation than the once-daily exercise, which in turn helps improve endurance performance.

A landmark study by Stensvold et al. [94], published in 2020, compared the effects of five years of supervised exercise training, including HIIT and MICT, with recommendations for physical activity on mortality in older adults [94]. A total of 1567 older adults were randomized into one of three groups: twice-a-week high-intensity interval exercise sessions (4 × 4 min at 85–95% peak HR (HR_peak_) with 3 min active recovery 60–70% HR_peak_); twice-a-week MICT sessions (50 min of continuous cycling at 70% HR_peak_); and a control group of generally active people who followed the Norwegian physical activity guidelines recommending 30 min of moderate-level physical activity almost every day without supervision [94]. After 5 years of follow-up, there were no superior effects of MICT and HIIT on all-cause mortality compared with recommended physical activity levels. Additionally, almost all of the 1567 participants had substantially lower mortality rates (around 5%) than expected in the age group (10%) [94], showing that exercise is essential for longevity. Further to this, the HIIT group showed the lowest mortality rates (3%), compared to the generally active group (4.7%) and the moderate-exercise group (5.9%), with no difference among the groups [94]. This study is the largest and longest randomized exercise study of that age group to date. Collectively, although this study does not appreciably prove that HIIT improves longevity, health authorities should be encouraged to recommend regular exercise for older adults, especially given that regular exercise is a relatively cheap and accessible treatment that can benefit a large proportion of the population.

### 3.3. Studies on Exercise Capacity and Health in Clinical Populations

Despite the growing HIIT literature with studies conducted in healthy individuals, there are also some cornerstone research that investigated whether the interval training model could be applied safely by people with various diseases. In these studies, researchers questioned whether such interval exercise could help individuals with different health problems to overcome metabolic disorders. For example, an early study in 1975, by Kavanagh and Shephard [95], reported a substantial increase in aerobic power following one year of interval training consisting of running or jogging intervals (1/2 to 1 min), followed by 1 to 1–1/2 min of slow walking, in post-coronary patients with frequent exercise-induced angina attacks and who had a poor previous response to several months of continuous training.

In the mid-1990s, Katharina Meyer and her colleagues incorporated different HIIT protocols on patients with heart failure, and for the first time, recommended interval training model to be better suited to improve cardiac function and physical performance in patients with chronic congestive heart failure [88,116,117]. Similarly, in 2004, Rognmo and colleagues aimed to reveal if 10 weeks of HIIT resulted in a similar or greater increase in $\dot{V}$O_2max_ when compared to MICT in coronary artery disease patients, and they reported an average 17.9% increase in the HIIT group and 7.9% in the MICT group [96]. This paper has shown HIIT to be more effective in increasing aerobic capacity in cardiac patients, which were previously reported in trained and healthy subjects, indicating interval exercise is able to optimize the exercise component of rehabilitation programs. Another study by Wisløff et al. that supported the findings of Helgerud and colleagues was published in 2008; it aimed to ascertain the effects of MICT and HIIT (3 sessions/wk for 12 weeks) on variables associated with cardiovascular function and prognosis in patients with postinfarction heart failure [97]. Patients aged 75.5 ± 11.1 years were randomly assigned to the HIIT group (4 × 4 min 90% to 95% of HR_peak_, separated by 3 min of light exercise 50% to 70% of HR_peak_) or the MICT group (47 min at 70% of HR_peak_) [97]. The major findings of the study were that peak oxygen uptake ($\dot{V}$O_2peak_) increased 46% and 14% in the HIIT and MICT groups, respectively, while PGC-1α only increased by 47% in the HIIT group, which was associated with an increase in $\dot{V}$O_2peak_ [97]. Besides, SV and peak ejection velocity, assessed by standard Doppler in the left ventricular outlet tract, increased by 17% and 19%, respectively, only in the HIIT group, but no change occurred in systolic function for the MICT group [97]. Additionally, pro-B-type natriuretic peptide levels, markers of hypertrophy and the severity of heart failure, decreased by 40% in HIIT group but remained unchanged in the MICT group [97]. Overall, this study indicated that HIIT was more effective than MICT model in improving left ventricular systolic performance and aerobic capacity in elderly heart failure patients.

In 2010, a study by Whyte et al. conducted 2 weeks of an “all-out” protocol [41] and the obtained findings showed SIT to be effective in increasing $\dot{V}$O_2max_ and PPO in overweight and obese men. Besides, a decrease in waist circumference, systolic blood pressure, fasting insulin, and resting carbohydrate oxidation was reported [41], while resting fat oxidation was higher after 2 weeks of SIT [41]. Based on these findings, SIT was suggested for the first time as a potential exercise model to improve vascular and metabolic health in sedentary overweight/obese populations. Moreover, it is worth noting that exercise with high intensity is associated with a lower risk of developing coronary heart disease; however, the safety of high-intensity interval exercise was questioned until a landmark study from a Norwegian research group compared the cardiovascular risk of HIIT against moderate-intensity aerobic exercise in patients with coronary heart disease [98]. The authors reported a low risk from performing both types of exercise (2 nonfatal cardiac arrests per 46,364 h of high-intensity interval exercise versus 1 fatal cardiac arrest per 129,456 h of moderate-intensity exercise) and proved the safety of high-intensity interval exercise in this population [98].

Taken together, given that the physiological adaptations and health benefits of HIIT, patients with coronary heart disease and obesity can be encouraged to perform such exercise. It should be noted, however, that more work is needed to ascertain if the supramaximal model of interval exercise is safe for widespread recommendation in the clinical population. Therefore, studies capable of comparing this protocol with submaximal HIIT and MICT for fatal and nonfatal cardiac events during exercise will provide an important first step towards the utilization of the “all-out” protocol as an exercise strategy for the overweight/obese population.

### 3.4. Studies on the Effects of HIIT on Glucose Tolerance and Insulin Sensitivity

It is well documented that regular exercise increases tissue sensitivity to insulin, while inactivity reverses this process that, in turn, causes impaired glycemic control, risk of pancreatic β cell failure, and the development of T2D [118]. These evidence-based therapeutic benefits of exercise on insulin sensitivity have led to a great interest in this area of research. In this context, given that “lack of time” is the most commonly cited impediment to exercise in a variety of populations [119], HIIT, especially low-volume HIIT, has been considered an alternative to MICT to improve metabolic health and insulin sensitivity [120]. For example, Babraj et al. [45] reported for the first time that six sessions of all-out exercise based on an SIT program resulted in a decrease in plasma glucose, insulin, and non-esterified fatty acid concentration-time curves; additionally, there was a 23% improvement in insulin sensitivity in healthy young males [45], indicating that very short-term HIIT seems to provide sufficient stimulus for improvements in glycemic control in sedentary young adults. However, as the exercise protocol employed in this study [45] and other studies [121,122] is extremely demanding and may not be practical for some individuals, Little et al. [99] investigated if low-volume HIIT designed by the same research group would improve hyperglycemia in patients with T2D. They reported a decrease in hyperglycemia and higher glucose transporter type 4 protein content following 2 weeks of low-volume HIIT consisting of 10 × 60 s cycling bouts eliciting ~90% HR_max_ with 60 s resting periods [99], showing improved insulin sensitivity. Further, another study in 2016 by Gillen et al. [100] compared the effects of 12 weeks of SIT (10 min per session) with MICT (50 min per session) on exercise capacity, insulin sensitivity, and skeletal muscle mitochondrial adaptations in obese adults. At the end of the training interventions, there were similar improvements in $\dot{V}$O_2peak_, insulin sensitivity, and maximal activity of CS, despite a five-fold difference in time commitment between the two groups [100]. This was a comparison of SIT and MICT with the longest duration, proving the efficacy of brief, intense exercise for physiological adaptations and indices of cardiometabolic health. Indeed, the findings of these studies are supported by meta-analyses [44,123,124,125] that have appreciably proven the effectiveness of HIIT on glycemic control and insulin sensitivity in healthy people and patients with T2D. Indeed, in 2020, Saner et al. questioned if a single bout of high-intensity interval exercise would mitigate sleep-loss-induced reductions in glucose tolerance, mitochondrial respiratory function, and sarcoplasmic protein synthesis [126]. A total of 24 young males were randomly assigned one of three groups: normal sleep group (8 h in bed per night, for five nights), a sleep-restriction group (4 h in bed per night, for five nights), and sleep restriction and exercise group (4 h in bed per night, for five nights and three high-intensity interval exercise sessions) [126]. The exercise protocol consisted of 10 × 60 s 90% PPO, separated by 75 s active recovery at 60 W (~25 min/per session, including the active, resting, and warm-up phases). The authors reported that glucose tolerance, maximal coupled mitochondrial respiration, and sarcoplasmic protein synthesis significantly reduced in the sleep-restriction group, but these perturbations were not observed in the sleep-restriction and exercise group [126]. This study indicates for the first time that the detrimental effects of sleep loss on glucose metabolism and mitochondrial functions and glucose tolerance can be overcome by performing a single bout of high-intensity interval exercise. However, it should be noted that this study did not involve a normal sleep and exercise group that could have elucidated the contribution of high-intensity interval exercise to overcome the negative metabolic effects of sleep restrictions. Further, it is still unknown if the observed benefits in response to high-intensity interval exercise in this study can be observed following 25 min of moderate- and high-intensity continuous exercise. Notably, a recent study by Flockhart et al. [101] investigated the effects of excessive 14 HIIT sessions (5 × 4–8 min of cycling at 95% of $\dot{V}$O_2max_, with 3 min of non-pedaling rest) over 4 weeks on mitochondrial and glucose tolerance in healthy volunteers (six women and five men). Training load for each participant was progressively increased until the fourth week, during which time the load was reduced to allow for recovery. At the end of week 3 (the highest training load), intrinsic mitochondrial respiration was markedly decreased, which coincided with a reduction in glucose tolerance and insulin secretion. There was a significant increase in physical performance and $\dot{V}$O_2max_ throughout the study, regardless of the training load [101]. This study exposes gaps in our current understanding of an upper limit to the amount of HIIT required to improve metabolic health before disruptions to mitochondrial function and whole-body metabolic homeostasis.

Taken together, the interval training program consisting of all-out, low- and high-volume HIIT protocols has been shown to be a time-efficient exercise strategy to improve insulin sensitivity and glucose tolerance. However, from a health perspective, exercise training programs should be carefully administrated, allowing the training response to be monitored, as too much exercise might result in negative effects. Besides, invasive methods such as glucose tolerance or careful tracking of glucose homeostasis might be easy approaches to optimize the amount of exercise associated with the greatest benefits.

## 4. Physiological Mechanisms Associated with HIIT-Induced Adaptations

### 4.1. Adaptations in $\dot{V}$O_2max_ and Endurance Capacity

The increase in aerobic capacity following exercise program depends on central and peripheral adaptations, including an increased capacity of the central nervous system to recruit motor units, increased SV, maximal cardiac output, blood flow, skeletal muscle mitochondrial content, and capillary density [127,128]. The magnitude of these adaptations depends on the intensity, duration, and frequency of exercise.

#### 4.1.1. Effects on $\dot{V}$O_2max_

**Figure 3:**
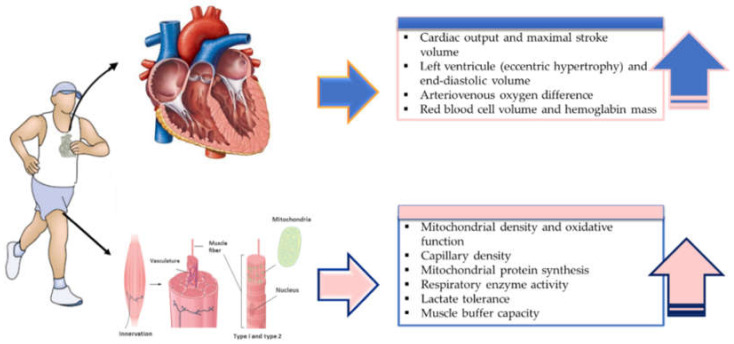
Central and peripheral adaptations to exercise training.

Meta-analysis studies have shown that despite lower training volume, HIIT results in a similar [129] or even greater [25,130,131,132] increase in $\dot{V}$O_2max_, in different populations including adolescents, healthy adults and people with obesity, cancer, and metabolic syndrome, compared to MICT, showing HIIT to be a time-efficient intervention for improving aerobic capacity in comparison to MICT. This observed increase in $\dot{V}$O_2max_ is usually attributed to increased SV [113], maximal cardiac output [29,133], maximal a-vO_2_ diff [127,134], skeletal-muscle oxidative enzyme capacity [70,122], capillary density [134], increased red blood cell volume, and hemoglobin mass [135], resulting in higher oxygen carrying capacity (Figure 3).

A meta-analysis by Sloth et al. that reviewed 13 studies, reported that improved aerobic performance and $\dot{V}$O_2max_ are associated with the peripheral adaptations following SIT, i.e., increased oxidative capacity of the muscle; however, current evidence regarding central adaptations is equivocal [136]. Another meta-analysis by Vollaard and colleagues [137] reported that improvement in $\dot{V}$O_2max_ following SIT was not attenuated with fewer sprint repetitions, clearly indicating that designing SIT interventions with fewer repetitions may offer health benefits with a lower exercise volume and a less demanding nature.

Although various studies have documented improved $\dot{V}$O_2max_, with concomitant peripheral adaptations in response to different HIIT and SIT programs [40,44,75,98,125], the cardiovascular adaptations to interval training have been examined in few studies [133,134,138,139] and remain poorly understood. For example, a study by Raleigh et al. [134] employed 8 × 20 s of pedaling at 170% of $\dot{V}$O_2max_, interspersed by 10 s of rest (4 days/week for 6 weeks), and reported a 9% increase in $\dot{V}$O_2max_ but no change in maximum cardiac output. Macpherson et al. assessed the effects of SIT versus MICT (3 times/week for 6 weeks) on 2000 m running time trial performance, $\dot{V}$O_2max_, and maximal cardiac output and reported similar improvements in time trial performance and $\dot{V}$O_2max_ in both groups [134], whereas maximal cardiac output increased only with MICT [140], showing that physiological adaptations following MICT are mainly central in origin, whereas those with SIT are more peripheral [140]. The findings of these two studies [134,140] suggest that peripheral adaptations, such as muscle oxidative enzymes, capillary density, and maximum a-vO_2_ diff play an essential role in the improvement in $\dot{V}$O_2max_ following interval training. In contrast, Astorino et al. [133] demonstrated an increase in maximal cardiac output but not in HR_max_ or a-vO_2_ diff following 6 weeks of HIIT, which consisted of 10 high-intensity interval exercise sessions (8 to 10 × 60 s at 90% to 110% PPO), followed by SIT, high-intensity interval exercise, or periodized interval training for the subsequent 10 sessions. All the training groups showed increased $\dot{V}$O_2max_, mediated by increases in SV [133]. In support of this finding, Warburton et al. showed that increase in $\dot{V}$O_2max_ following 12 weeks of HIIT was mediated by increased SV and increases in vascular volumes [141]. Others documented improvements in $\dot{V}$O_2max_ attributable to both central and peripheral adaptations [29,142]. De Revere at al. [142] showed that nine sessions of HIIT over 3 weeks provided the sufficient level of stimulus to increase cardiac output, with a concomitant increase in $\dot{V}$O_2max_. The authors also reported a trivial increase in a-vO_2_ diff indicating the possibility that a-vO_2_ diff plays an important role in increased $\dot{V}$O_2max_ following exercise. Similarly, Daussin et al. reported that 8 weeks of interval training resulted in improved $\dot{V}$O_2max_, along with increases in both peripheral muscle and central adaptations in inactive adults [29]. These discrepancies in the published literature are likely due to differences in training protocols, study durations and populations, or total work done.

#### 4.1.2. Effects on Endurance Capacity

It is well-documented that HIIT administered for different durations is an effective way to enhance exercise performance [38,70,122,143]. Mostly determined using time-to-exhaustion or time trials, HIIT-induced improvements in endurance capacity are attributed to both molecular and physiological factors, including increased maximal CS activity [128], skeletal-muscle blood flow and vascular conductance [144], lactate carrying capacity and hydrogen ion release capacity from active muscles [145], and sarcoplasmic reticulum function [146]. Besides, the increased $\dot{V}$O_2max_ and oxidative capacity of skeletal muscle following HIIT play a major role in improving exercise performance [91,127], allowing the same exercise intensity to be performed for longer after the training intervention. It is also worth noting that one of the hallmark adaptations following SIT or HIIT programs that affects exercise performance is increased fat oxidation during exercise, resulting in less accumulation of intracellular metabolites (lactate, hydrogen ion, inorganic phosphate, adenosine diphosphate, and adenosine monophosphate) and the sparing of glycogen stores [18,22].

### 4.2. Skeletal Muscle Adaptations to HIIT

Exercise is traditionally classified as being either aerobic/endurance or strength/resistance [147]. The nature of the physiological adaptations to exercise is associated with the exercise protocols applied, which activate different molecular pathways. For example, endurance training models, including HIIT, provide physiological stimuli for mitochondrial biogenesis, which in turn reduces glycogen use and lactate production, increases the lactate threshold, and allows individuals to exercise longer at a given intensity [148,149]. On the other hand, strength training stimulates the myofibrillar protein synthesis leading to muscle hypertrophy and increased maximal strength. In this context, the interesting aspect of HIIT is that this type of training promotes physiological adaptations known to be induced by MICT, yet the pattern of short, vigorous repetitions separated by light exercise is similar to resistance exercise.

**Figure 4:**
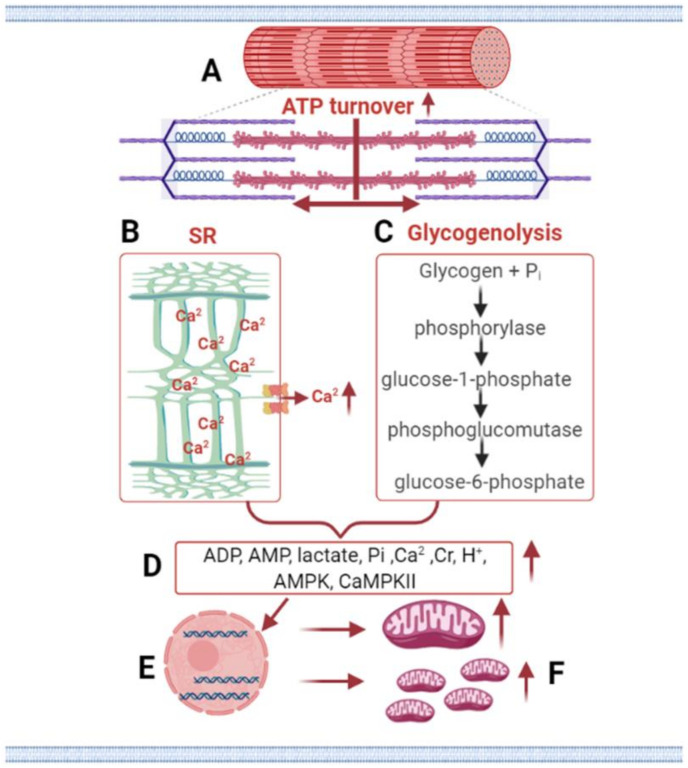
Schematic of the main signaling pathways through which high-intensity exercise elicits greater mitochondrial adaptations compared to lower intensities of exercise. Exercising at a higher intensity requires greater adenosine triphosphate turnover (**A**) and increases calcium release from sarcoplasmic reticulum; (**B**) carbohydrate oxidation, particularly from muscle glycogen, dominates at higher exercise intensities, compared to exercising at a lower intensity. (**C**) This results in a greater accumulation of metabolites, such as adenosine diphosphate, adenosine monophosphate, lactate, inorganic phosphate, creatine, calcium, hydrogen ion, adenosine monophosphate-activated protein kinase, and calcium/calmodulin-dependent protein kinase II, (**D**) causing greater rates of gene expression, (**E**) which promotes greater mitochondrial protein synthesis rates and greater mitochondrial content. (**F**) ADP, adenosine diphosphate; AMP, adenosine monophosphate; AMPK, adenosine monophosphate-activated protein kinase; ATP, adenosine triphosphate; Ca^2^, calcium; CaMPKII, calcium/calmodulin-dependent protein kinase II; Cr, creatine; H^+^, hydrogen ion; Pi, inorganic phosphate; SR; sarcoplasmic reticulum.

Although studies having investigated how HIIT provides similar or greater adaptations to MICT in a much shorter time [72,87,100], knowledge remains limited regarding the physiological mechanisms induced by interval training, and the communication between metabolic pathways within cells. During one hour of moderate-intensity exercise, oxygen supply is adequate, and the substrate demand of active muscles is largely met by the oxidation of carbohydrate and fats. Type I muscle fibers are predominantly used, and the rate of change in whole-body steady-state is trivial and maintained. On the other hand, during high-intensity continuous or interval exercise that exceeds the threshold stimulus, widespread disturbances occur to both local (muscular) and systemic (cardiovascular, respiratory, neural and hormonal) steady-state. Mostly type II muscle fibers are actively used, which increases ATP production up to 100 times to meet the increased energy demand of the muscle [147,148]. This subsequently increases intracellular lactate, phosphocreatine, adenosine monophosphate, and adenosine diphosphate accumulation, as well as the activity of adenosine monophosphate-activated protein kinase and calmodulin-dependent protein kinase II. The increased activation of these kinases induces higher messenger RNA (mRNA) expression of PGC-1α, considered the master regulator of mitochondrial biogenesis [150]. As a result of these physiological processes and cellular stress, which occur in proportion to the intensity of endurance exercise [60], mitochondrial protein synthesis, and mitochondrial biogenesis are observed [17,93,151,152,153,154] (Figure 4).

Physiological adaptations in response to interval training are dependent on not only the intensity of exercise but also the subsequent rest intervals [19]. A study supporting this notion showed that 30 min of intermittent moderate-intensity exercise (30 × 1 min intervals, separated by 1 min of recovery) induces higher (~2.9 fold) activation of AMP-activated protein kinase (AMPK), which regulates PGC-1α, compared to a single bout of 30 min of continuous exercise [155]. This finding indicates that intermittent exercise protocols are likely to induce greater mitochondrial adaptation than acute moderate-intensity continuous exercise [17,19]. However, there are some limitations to this study that should be considered when interpreting the results. First, even though effort time was similar, a single bout of high-intensity interval exercise led to greater session duration (60 min versus 30 min) that goes against the idea of being time-effective. Besides, muscle biopsies were taken upon the cessation of cycling, meaning 30 min after the beginning of continuous exercise but 60 min after the onset of the high-intensity interval exercise session. Further, since no time-course of signaling protein activation was provided, which would be difficult to do due to the invasive nature of the procedure, this could be a confusing factor that limits the findings of this study. Considering these limitations, the question that remains unanswered is whether the activation of AMPK would be the same when compared between both conditions at 30 min and at 60 min rather than 30 min versus 60 min. In this context, it is apparently a fertile area of research to uncover whether these acute changes observed in signaling patterns in response to a single bout of high-intensity interval exercise could translate to different chronic effects.

### 4.3. Adaptations to Once- or Twice-Daily HIIT

Most studies that existed in the literature consist of HIIT protocols that were performed once daily, every other day [44,47,75,98,100,125]. However, some studies also investigated the physiological effects of HIIT performed twice-a-day [93]. For example, it has recently been shown that 6 high-intensity interval exercise sessions performed twice-a-day over just 5 days markedly improved $\dot{V}$O_2max_, endurance capacity, and submaximal exercise fat oxidation [38]. This study is the shortest HIIT study to provide sufficient stimulus for such adaptations in response to a HIIT regime and represents an important conceptual advancement for training prescription, demonstrating the remarkable ability of the human body to adapt to exercise stress in less than 1 week. In contrast to the “once-daily” exercise approach, the number of the published papers on the effects of twice-a-day HIIT on performance variables, energy metabolism, and possible molecular mechanisms is limited [93,156,157,158,159,160,161]. Some researchers have investigated the physiological effects of the twice-a-day approach on mitochondrial adaptations, measuring mitochondrial biogenesis and mitochondrial efficiency [93,158]. These studies reported twice-daily exercise sessions (wherein the second session was commenced with reduced muscle glycogen stores) improved energy metabolism and induced skeletal-muscle cell-signaling pathways regulating mitochondrial and substrate metabolism [159,160,161,162]. Of the limited studies available, the twice-a-day approach has also been shown to be more effective than “once-daily” exercise for inducing adaptations related to substrate oxidation during submaximal exercise, endurance capacity, and $\dot{V}$O_2max_ [3,156,160,161]. In these studies, the first exercise sessions were generally an exercise that depleted muscle-glycogen stores, and the second exercise session was performed with low muscle-glycogen stores that potentiated the exercise-induced increase in genes linked to mitochondrial biogenesis and metabolism [156,160,161,163,164,165]. The main findings of these studies suggest that a single bout of high-intensity interval exercise performed after a muscle-glycogen-depleting exercise session induced greater mitochondrial adaptations, improved endurance capacity (as measured by time-to-exhaustion or time-trial performance), and whole-body substrate oxidation [156,160,161]. The putative mechanisms involved are hard to define. In the light of the available evidence, however, it is tempting to speculate that these adaptations are not associated with low glycogen availability. Instead, HIIT with low glycogen stores may elicit higher perturbation in steady-state and higher increase in mitochondrial volume [161], when compared to HIIT with normal glycogen stores, resulting in higher training adaptation and endurance performance. Further, Hansen et al. [166] reported a higher catecholamine response to HIIT performed at low muscle-glycogen stores than at exercise performed at high muscle-glycogen stores, indicating higher stress response when the muscle glycogen is low, whilst Hulston et al. [163] reported that exercising with low muscle glycogen was not more effective for training adaptation than with high muscle glycogen in already well-trained athletes. Taken together, two workouts in close proximity, with the second bout of exercise performed at low muscle-glycogen content, seems to be a time-efficient method of maintaining training adaptations and performance, especially for untrained individuals. However, it should be noted that training with a high muscle-glycogen content would allow one to train for longer periods and thus obtain better results. Moreover, training programs aiming at decreasing muscle glycogen may lead to the risk for the so-called overtraining syndrome and impaired immune function, which hinder performance improvement.

## 5. Conclusions

In this paper, a brief history of interval training was presented, based on the novel findings of some selected studies on exercise capacity and health, starting from the early 1920s to date. Furthermore, an overview of the mechanisms underlying the physiological adaptations in response to interval training was provided.

It is well-documented that regular exercise is essential for a healthy life, but insufficient time to exercise seems to be one of the most cited barriers to exercise adherence. Considerable evidence has shown that interval training models can provide similar health and performance benefits to MICT, despite less time commitment. It is also apparent that different interval training models, including HIIT and SIT, are effective health- and performance-enhancing exercise strategies. Further, despite similar time commitment required for some high-intensity interval exercise models and continuous exercise, current evidence shows that high-intensity interval exercise can elicit higher enjoyment and greater physiological adaptation than MICT, making HIIT an effective strategy for a regular exercise habit. In addition, given that excessive HIIT intervention might result in detrimental metabolic effects as recently reported, monitoring exercise intensity carefully is of primary importance for the above-mentioned benefits of this type of exercise. Further randomized controlled trials involving long-term interventions (≥12 weeks) are warranted to determine whether low-volume HIIT offers similar or greater health and performance benefits when compared to MICT and high-volume HIIT in healthy individuals and those at risk of chronic-inactivity-related diseases. It should also be noted that most studies included men and healthy people; thus, the findings of these studies cannot be applied to women and patients with different chronic diseases. Moreover, the feasibility and safety of supramaximal interval models have not been addressed in patients with cardiac disorders, who are at an increased risk for sudden cardiac arrest during vigorous physical exercise compared to healthy individuals [167]. Therefore, further works are needed to investigate the feasibility and safety of SIT based on clinical characteristics and fitness level. Although people not engaging in regular physical activity have a greater risk of myocardial infarction during or soon after exertion [168], and the incidence of sudden cardiac arrest across a variety of activities is similar to that expected by chance alone [167], more studies are warranted to assess the safety of SIT in the clinical population. Additionally, twice-daily high-intensity interval exercise interventions remain fruitful areas of investigation to uncover possible physiological mechanisms underlying improved cardiorespiratory fitness with briefer training program duration. Further studies along with the findings of the published studies will help promote greater incorporation of HIIT into daily life and training program. These works will also open up new avenues to help translate these types of exercise models into physical activity recommendations for the general population.

## Figures and Tables

**Table 1:** Description of some selected HIIT studies.

	Author	Year	Participants ($\dot{V}$O_2_max)(mL/kg/min)	*n* (M/F)	Duration; Frequency; Mode	Protocols	Main Findings
1	Knuttgen et al. [82]	1973	Active male (~45.3 $\dot{V}$O_2_max)	(60/0)	1–2-months; 3–5 days/week; cycling	**Group 1:** 15 s all-out and 15 s rest 3 days/week for 2 months **Group 2:** 3 min at $\dot{V}$O_2_max and 3 min rest, 3 days/week for 2 months **Group 3:** 15 min of strenuous exercise/sessions, 5 days/week for 1 month	Increase in $\dot{V}$O_2_max, with a concomitant reduction in HR at submaximal exercise. **$\dot{V}$O_2_max (mL/kg/min)** **Group 1:** 45.8 to 52.6 **Group 2:** 43.1 to 53.4 **Group 3:** 46.4 to 57.0
2	Fox et al. [83]	1975	Young, healthy male (~45.5 $\dot{V}$O_2_max)	(69/0)	7–13-weeks; 2–4 days/week; running	**Group 1:** 2-day of short- (50–201 m), 1 day of long- (604–1208 m), 1 day of both short- and long-distance running (4 days/week for 7 weeks) **Group 2:** 1 day of long-, 1 day of both short- and long-distance running (2 days/week for 7 weeks) **Group 3:** 2-day of short-, 1 day of long-, 1 day of short- and long-distance running (4 days/week for 13 weeks) **Group 4:** 1 day of long-, 1 day of both short- and long-distance running (2 days/week for 13 weeks)	Increase in $\dot{V}$O_2_max, with no difference between the change due to training, training frequency, or training duration. Similar decrease in HRmax in all groups. **$\dot{V}$O_2_max (mL/kg/min)** **Group 1:** 43.5 to 48.0 **Group 2:** 44.2 to 48. **Group 3:** 43.2 to 49.2 **Group 4:** 41.9 to 47.7
3	Henriksson and Reitman [84]	1976	Young, healthy male (51.5 $\dot{V}$O_2_max)	(9) NS	7–8-weeks; 3 days/week; cycling	**Group 1:** 5 × 4 min at 101% $\dot{V}$O_2_max, separated by 2 min rest **Group 2:** 27 min of continuous exercise at 79% of $\dot{V}$O_2_max	Increase in maximal activities of SDS in both groups.
4	Roberts et al. [85]	1982	Active male (NR)	(4/0)	5-weeks; 3–4 days/week; running	16 sessions of high-intensity interval exercise consisting of eight 200 m run at 90% of the maximal speed (HR ~179 beats/min), separated by 2 min rest periods (HR ~130 beats/min)	Increase in glycolytic enzymes (GAPDH, LDH, MDH, PFK), as well as endurance capacity (~20%). determined by a treadmill test at 16 km/h, 15% grade to exhaustion.
5	Sharp et el. [86]	1986	Young, healthy male (~52.7 $\dot{V}$O_2_max)	(15/0)	8-weeks; 4 days/week; cycling	8 × 30 s all-out with 4 min of rest	Increase in $\dot{V}$O_2_max, buffer capacity, and activity of PFK.
6	Tabata et al. [87]	1996	Young male (50.5 $\dot{V}$O_2_max)	(14/0)	4–6-weeks; 5 days/week; cycling	**Group 1:** 7–8 × 20 s with 10 s rest (4 days/week)—30 min of cycling at 70% and 4 × 20 s at 170% $\dot{V}$O_2_max (1 day/week) **Group 2:** 60 min of continuous exercise at 70% $\dot{V}$O_2_max	Increase in $\dot{V}$O_2_max (10–15%) in both groups and concomitant increase in anaerobic capacity only in interval group.
7	Meyer et al. [88]	1990	Patients having undergone coronary bypass surgery (NR)	(18/0)	3.5-weeks; 7 days/week; cycling	**Group 1:** 20–25 × 1 at 86% of HRmax, separated by 1 min of recovery at 20 W **Group 2:** 20–25 min of continuous exercise at 86% of HRmax	Increase physical performance and economization of cardiac function, as well as larger decrease in HR at rest and during exercise, in the interval group.
8	MacDougall et al. [89]	1998	Young, healthy men (47.8 $\dot{V}$O_2_max)	(20/0)	7-weeks; 3 days/week; cycling	4–10 × 30 s all-out with 2–4 min of recovery.	Increase in $\dot{V}$O_2_max, endurance capacity, and glycolytic and oxidative enzyme activity.
9	Gibala et al. [71]	2006	Active male (50.9 $\dot{V}$O_2_max)	(16/0)	2-weeks; 3 days/wk; cycling	**Group 1:** 4–6 × 30 s at ~250% $\dot{V}$O_2_peak with 4 min recovery (Total time commitment: 2.5 h) **Group 2:** 90–120 min of continuous exercise 70% $\dot{V}$O_2_max (Total time commitment: ~10.5 h)	Similar increase in time to trial performance, muscle buffering capacity, and glycogen content in both groups despite markedly less time commitment in group 1.
10	Helgerud et al. [90]	2007	Trained male (57.9 $\dot{V}$O_2_max)	(40/0)	8-weeks; 3 days/wk; running	**Group 1:** 45 min of running at 70% HRmax **Group 2:** 25 min of running at 70% HRmax **Group 3:** 47 × 15 s interval at 90–95% HRmax with 15 s active resting periods **Group 4:** 4 × 4 min at 90–95% HRmax with 3 min active resting periods at 70% HRmax	Similar increase in $\dot{V}$O_2_max and SV only in group 3 and group 4.
11	Little et al. [91]	2010	Young, healthy men (46.0 $\dot{V}$O_2_peak)	(7/0)	2-weeks; 3 days/week; cycling	8–10 × 1 min at ~100% HRpeak with 75 s recovery	~10.0%, ~18%, 29%, ~24%, ~56%, ~119 and 17% increase in endurance capacity, CS, COX, PGC-1α, SIRT1, glucose transporter type 4, and resting muscle glycogen, respectively.
12	Granata et al. [92]	2016	Young, healthy men (46.3 $\dot{V}$O_2_peak)	(29/0)	4-weeks; 3 days/week; cycling	**Group 1:** 4–10 × 30 s all out with a 2 min rest **Group 2:** 4–7 × 4 min at 90% $\dot{V}$O_2_peak with 2 min recovery at 60 W **Group 3:** 20–36 min at ~90% $\dot{V}$O_2_peak	Improved endurance capacity only in group 2 and 3; increase in PGC-1α protein content and mitochondrial respiration only in group 1.
13	Granata et al. [93]	2016	Young, healthy men (45.1 $\dot{V}$O_2_peak)	(10/0)	14-weeks; 3 days/week; cycling	**3 consecutive training programs** **Program 1:** normal volume training, involving 4–7 × 4 min with a 2 min recovery at 60 W (3 times/week for 4 week) **Program 2:** high volume training, twice a day for 20 consecutive days, involving 5–12 × 4 min intervals or 8–22 × 2 min intervals with a 1 min recovery at 60 W **Program 3:** 1 and 4 sessions of 4 × 4 min and 1–5 × 2 min, respectively, for 2 weeks	Increase in $\dot{V}$O_2_max, endurance performance, mitochondrial content, and mitochondrial respiration following high volume HIIT, and these gains returned to baseline after 2 week of reduced volume training.
14	Stensvold et al. [94]	2020	Older adults (28.7 $\dot{V}$O_2_peak)	(777/790)	12-weeks; 2 days/week; cycling	**Group 1:** 4 × 4 min at 85–95% HRpeak with 3 min active recovery 60–70% HRpeak **Group 2:** 50 min of continuous cycling at 70% HRpeak **Group 3:** National recommendation (30 min of moderate-level physical activity every day without supervision)	Higher increase in $\dot{V}$O_2_max and physical component continuous summary score in group 1 than the other groups. No effect on all-cause mortality in group 1 and 2 compared with recommended physical activity levels.
15	Kavanagh and Shephard [95]	1975	Postcoronary patients (NR)	(41/0)	1 year; 5 days/week; running	**Group 1:** 24–30 min of continuous training at 60–70% $\dot{V}$O_2_max. **Group 2:** 10–30 × 1 min of jogging or running at 75% of difference resting HR and HRmax, separated by 1 min of recovery at 40% difference resting HR and HRmax.	Substantial increase in aerobic power calculated based on work and oxygen of the Astrand scale in both groups, with higher gains in patients suffering frequent angina, following interval training.
16	Rognmo et al. [96]	2004	Coronary artery disease patients (31.9 $\dot{V}$O_2_peak)	(14/3)	10-weeks; 3 days/week; running	**Group 1:** 4 × 4 min at 80–90% HRmax with 3 min active resting periods at 60% $\dot{V}$O_2_peak **Group 2:** 41 min of continuous running at 50–60% $\dot{V}$Opeak	17.9% and 7.9% increase in $\dot{V}$O_2_max in group 1 and group 2, respectively.
17	Wisløff et al. [97]	2008	Postinfarction heart failure patients (13.1 $\dot{V}$O_2_peak)	(20/7)	12-weeks; 3 days/week; running	**Group 1:** 4 × 4 min at 90–95% HRpeak with 3 min active resting periods at ~60% HRpeak **Group 2:** 47 min of continuous running at 70–75% HRpeak **Group 3:** No exercise	46.0% and 14.0% increase in $\dot{V}$O_2_max in group 1 and group 2, respectively, and a 47% increase in PGC-1α only group 1.
18	Whyte et al. [41]	2010	Overweight and obese men (32.8 $\dot{V}$O_2_peak)	(10/0)	2-weeks; 3 days/week; cycling	4–6 × 30 s all out with 4.5 min recovery at 30 W	8.4% and 18.2 increase in $\dot{V}$O_2_max and resting fat oxidation, respectively, and 24.5% and 4.7 decreases in fasting insulin and systolic blood pressure, respectively.
19	Rognmo et al. [98]	2012	Coronary heart disease patients (NR)	(3393/1453)	-	**Group 1:** 4 × 4 min at 85–95% HRpeak with 3 min active resting periods at ~60% HRpeak **Group 2:** 47 min of continuous running at 60–70% HRpeak	1 nonfatal cardiac arrest during high-intensity interval exercise per 23,182 exercise hours, 1 fatal cardiac arrest during MIT per 129,456 exercise hours.
20	Babraj et al. [45]	2009	Young, healthy men (48.0 $\dot{V}$O_2_peak)	(16/0)	2-weeks; 3 days/week; cycling	**Group 1:** 4–6 × 30 s all out with 4 min recovery at 30 W **Group 2:** No exercise	23% and 6% improvements in insulin sensitivity, endurance capacity, and reduced fasting plasma NEFA concentrations.
21	Little et al. [99]	2011	Patients with type 2 diabetes (NR)	(8) NS	2-weeks; 3 days/week; cycling	10 × 1 min at ~90% HRpeak with 60 s rest	Reduced blood glucose concentration and improved glucose transporter type 4 protein content, muscle mitochondrial capacity, and the maximal activity of CS.
22	Gillen et al. [100]	2016	Sedentary men (32.5 $\dot{V}$O_2_peak)	(25/0)	12-weeks; 3 days/week; cycling	**Group 1:** 3 × 20 s all-out with 3 min recovery at 50 W **Group 2:** 45 min of continuous cycling at 70% $\dot{V}$O_2_peak **Group 3:** No exercise	Similar increase in $\dot{V}$O_2_max, insulin sensitivity, and mitochondrial content and the maximal activity of CS in intervention groups, despite a five-fold lower exercise-volume required in group 1.
25	Flockhart et al. [101]	2021	Young, healthy men and women (48.4 $\dot{V}$O_2_max)	(5/6)	4 weeks; progressively increased work load; cycling	**14 HIIT-sessions in total (~95% of VO_2_max)** **1st week:** 2 × 5 × 4-min **2nd week:** 2 × 5 × 8-min & 1 × 5 × 4-min **3rd week:** 3 × 5 × 8-min & 2 × 5 × 4-min **4th week:** 2 × 3 × 8-min & 1 × 3 × 4-min & 1 × 1 × 4-min	**At the end of the** **1st week and 2nd week:** Unaltered glucose AUC and improved PPO. **3rd week:** Reduction in mitochondrial intrinsic respiration, glucose tolerance, AUC for plasma insulin, HOMA-β, and higher increase in lipid oxidation compared to 1st and 2nd week. **4th week (recovery):** Partly and fully recovered glucose tolerance and HOMA-β, respectively.

AUC; area under the curve, COS; cytochrome C oxidase, CS; citrate synthase, F; female, GAPDH; glyceraldehyde phosphate dehydrogenase, The HOMA; homeostasis model assessment, HR; heart rate, HRmax; maximal heart rate, HRpeak; peak heart rate, LDL; lactate dehydrogenase, M; male, MDH; malate dehydrogenase, MIT; moderate-intensity training, NEFA; non-esterified fatty acid, NR; not reported, NS; not specified, PGC-1α; peroxisome proliferator-activated receptor gamma coactivator 1 alpha, PFK; phosphofructokinase, SDH; succinate-dehydrogenase, $\dot{V}$O_2_max; maximal oxygen uptake; $\dot{V}$O_2_peak; peak oxygen uptake; W; watt.

## Data Availability

Not applicable.

## References

[1] Booth F.W., Roberts C.K., Laye M.J. (2012). Lack of exercise is a major cause of chronic diseases. Compr. Physiol..

[2] Warburton D.E., Nicol C.W., Bredin S.S. (2006). Health benefits of physical activity: The evidence. CMAJ.

[3] Atakan M.M., Kosar S.N., Guzel Y., Tin H.T., Yan X. (2021). The Role of Exercise, Diet, and Cytokines in Preventing Obesity and Improving Adipose Tissue. Nutrients.

[4] Sylow L., Richter E.A. (2019). Current advances in our understanding of exercise as medicine in metabolic disease. Curr. Opin. Physiol..

[5] Febbraio M.A. (2017). Exercise metabolism in 2016: Health benefits of exercise - more than meets the eye!. Nat. Rev. Endocrinol..

[6] Murphy R.M., Watt M.J., Febbraio M.A. (2020). Metabolic communication during exercise. Nat. Metab..

[7] Azadpour N., Tartibian B., Kosar S.N. (2017). Effects of aerobic exercise training on ACE and ADRB2 gene expression, plasma angiotensin II level, and flow-mediated dilation: A study on obese postmenopausal women with prehypertension. Menopause.

[8] Pedisic Z., Shrestha N., Kovalchik S., Stamatakis E., Liangruenrom N., Grgic J., Titze S., Biddle S.J., Bauman A.E., Oja P. (2020). Is running associated with a lower risk of all-cause, cardiovascular and cancer mortality, and is the more the better? A systematic review and meta-analysis. Br. J. Sports Med..

[9] Colberg S.R., Sigal R.J., Fernhall B., Regensteiner J.G., Blissmer B.J., Rubin R.R., Chasan-Taber L., Albright A.L., Braun B., American College of Sports M. (2010). Exercise and type 2 diabetes: The American College of Sports Medicine and the American Diabetes Association: Joint position statement. Diabetes Care.

[10] Assi M., Dufresne S., Rébillard A. (2020). Exercise shapes redox signaling in cancer. Redox Biol..

[11] Lee S.W., Jo H.H., Kim M.R., You Y.O., Kim J.H. (2012). Association between obesity, metabolic risks and serum osteocalcin level in postmenopausal women. Gynecol. Endocrinol..

[12] Hallal P.C., Andersen L.B., Bull F.C., Guthold R., Haskell W., Ekelund U. (2012). Global physical activity levels: Surveillance progress, pitfalls, and prospects. Lancet.

[13] WHO (2020). Guidelines on Physical Activity and Sedentary Behaviour.

[14] Bull F.C., Al-Ansari S.S., Biddle S., Borodulin K., Buman M.P., Cardon G., Carty C., Chaput J.-P., Chastin S., Chou R. (2020). World Health Organization 2020 guidelines on physical activity and sedentary behaviour. Br. J. Sports Med..

[15] Cassidy S., Thoma C., Houghton D., Trenell M.I. (2017). High-intensity interval training: A review of its impact on glucose control and cardiometabolic health. Diabetologia.

[16] Buchheit M., Laursen P.B. (2013). High-intensity interval training, solutions to the programming puzzle: Part I: Cardiopulmonary emphasis. Sports Med..

[17] Bishop D.J., Botella J., Genders A.J., Lee M.J., Saner N.J., Kuang J., Yan X., Granata C. (2019). High-Intensity Exercise and Mitochondrial Biogenesis: Current Controversies and Future Research Directions. Physiology.

[18] MacInnis M.J., Gibala M.J. (2016). Physiological adaptations to interval training and the role of exercise intensity. J. Physiol..

[19] Gibala M.J., Little J.P., Macdonald M.J., Hawley J.A. (2012). Physiological adaptations to low-volume, high-intensity interval training in health and disease. J. Physiol..

[20] Pattyn N., Beulque R., Cornelissen V. (2018). Aerobic Interval vs. Continuous Training in Patients with Coronary Artery Disease or Heart Failure: An Updated Systematic Review and Meta-Analysis with a Focus on Secondary Outcomes. Sports Med..

[21] Pescatello L.S. (2014). ACSM’s Guidelines for Exercise Testing and Prescription.

[22] Astorino T.A., Schubert M.M. (2018). Changes in fat oxidation in response to various regimes of high intensity interval training (HIIT). Eur. J. Appl. Physiol..

[23] Buchheit M., Laursen P.B. (2013). High-intensity interval training, solutions to the programming puzzle. Part II: Anaerobic energy, neuromuscular load and practical applications. Sports Med..

[24] Gibala M.J., Bostad W., McCarthy D.G. (2019). Physiological adaptations to interval training to promote endurance. Curr. Opin. Physiol..

[25] Weston K.S., Wisloff U., Coombes J.S. (2014). High-intensity interval training in patients with lifestyle-induced cardiometabolic disease: A systematic review and meta-analysis. Br. J. Sports Med..

[26] Girard O., Mendez-Villanueva A., Bishop D. (2011). Repeated-sprint ability—Part I: Factors contributing to fatigue. Sports Med..

[27] Gray S.R., Ferguson C., Birch K., Forrest L.J., Gill J.M. (2016). High-intensity interval training: Key data needed to bridge the gap from laboratory to public health policy. Br. J. Sports Med..

[28] Gibala M.J., Jones A.M. (2013). Physiological and performance adaptations to high-intensity interval training. Nestle Nutr. Inst. Workshop Ser..

[29] Daussin F.N., Zoll J., Dufour S.P., Ponsot E., Lonsdorfer-Wolf E., Doutreleau S., Mettauer B., Piquard F., Geny B., Richard R. (2008). Effect of interval versus continuous training on cardiorespiratory and mitochondrial functions: Relationship to aerobic performance improvements in sedentary subjects. Am. J. Physiol Regul Integr Comp. Physiol.

[30] Gorostiaga E.M., Walter C.B., Foster C., Hickson R.C. (1991). Uniqueness of interval and continuous training at the same maintained exercise intensity. Eur. J. Appl. Physiol. Occup. Physiol..

[31] Langan S.P., Grosicki G.J. (2021). Exercise Is Medicine…and the Dose Matters. Front. Physiol..

[32] Thum J.S., Parsons G., Whittle T., Astorino T.A. (2017). High-Intensity Interval Training Elicits Higher Enjoyment than Moderate Intensity Continuous Exercise. PLoS ONE.

[33] Oliveira B.R.R., Santos T.M., Kilpatrick M., Pires F.O., Deslandes A.C. (2018). Affective and enjoyment responses in high intensity interval training and continuous training: A systematic review and meta-analysis. PLoS ONE.

[34] Reljic D., Lampe D., Wolf F., Zopf Y., Herrmann H.J., Fischer J. (2019). Prevalence and predictors of dropout from high-intensity interval training in sedentary individuals: A meta-analysis. Scand. J. Med. Sci. Sports..

[35] Thompson W.R. (2017). Worldwide Survey of Fitness Trends For 2018. ACSM’s Health Fit. J..

[36] Thompson W.R. (2018). Worldwide Survey of Fitness Trends For 2019. ACSM’s Health Fit. J..

[37] Batacan R.B., Duncan M.J., Dalbo V.J., Tucker P.S., Fenning A.S. (2017). Effects of high-intensity interval training on cardiometabolic health: A systematic review and meta-analysis of intervention studies. Br. J. Sports Med..

[38] Atakan M.M., Güzel Y., Bulut S., Koşar N., McConell G.K., Turnagöl H.H. (2020). Six high-intensity interval training sessions over 5 days increases maximal oxygen uptake, endurance capacity, and sub-maximal exercise fat oxidation as much as 6 high-intensity interval training sessions over 2 weeks. J. Sport Health Sci..

[39] Rosenblat M.A., Perrotta A.S., Thomas S.G. (2020). Effect of High-Intensity Interval Training Versus Sprint Interval Training on Time-Trial Performance: A Systematic Review and Meta-analysis. Sports Med..

[40] Schubert M.M., Clarke H.E., Seay R.F., Spain K.K. (2017). Impact of 4 weeks of interval training on resting metabolic rate, fitness, and health-related outcomes. Appl. Physiol. Nutr. Metab..

[41] Whyte L.J., Gill J.M., Cathcart A.J. (2010). Effect of 2 weeks of sprint interval training on health-related outcomes in sedentary overweight/obese men. Metabolism..

[42] Talanian J.L., Galloway S.D., Heigenhauser G.J., Bonen A., Spriet L.L. (2007). Two weeks of high-intensity aerobic interval training increases the capacity for fat oxidation during exercise in women. J. Appl. Physiol. (1985).

[43] Sultana R.N., Sabag A., Keating S.E., Johnson N.A. (2019). The Effect of Low-Volume High-Intensity Interval Training on Body Composition and Cardiorespiratory Fitness: A Systematic Review and Meta-Analysis. Sports Med..

[44] Jelleyman C., Yates T., O’Donovan G., Gray L.J., King J.A., Khunti K., Davies M.J. (2015). The effects of high-intensity interval training on glucose regulation and insulin resistance: A meta-analysis. Obes. Rev..

[45] Babraj J.A., Vollaard N.B., Keast C., Guppy F.M., Cottrell G., Timmons J.A. (2009). Extremely short duration high intensity interval training substantially improves insulin action in young healthy males. BMC Endocr. Disord..

[46] Drigny J., Gremeaux V., Dupuy O., Gayda M., Bherer L., Juneau M., Nigam A. (2014). Effect of interval training on cognitive functioning and cerebral oxygenation in obese patients: A pilot study. J. Rehabil. Med..

[47] Hsieh S.S., Chueh T.Y., Huang C.J., Kao S.C., Hillman C.H., Chang Y.K., Hung T.M. (2021). Systematic review of the acute and chronic effects of high-intensity interval training on executive function across the lifespan. J. Sports Sci..

[48] Mekari S., Neyedli H.F., Fraser S., O’Brien M.W., Martins R., Evans K., Earle M., Aucoin R., Chiekwe J., Hollohan Q. (2020). High-Intensity Interval Training Improves Cognitive Flexibility in Older Adults. Brain Sci..

[49] Mijwel S., Jervaeus A., Bolam K.A., Norrbom J., Bergh J., Rundqvist H., Wengström Y. (2019). High-intensity exercise during chemotherapy induces beneficial effects 12 months into breast cancer survivorship. J. Cancer Surviv..

[50] Dun Y., Thomas R.J., Smith J.R., Medina-Inojosa J.R., Squires R.W., Bonikowske A.R., Huang H., Liu S., Olson T.P. (2019). High-intensity interval training improves metabolic syndrome and body composition in outpatient cardiac rehabilitation patients with myocardial infarction. Cardiovasc. Diabetol..

[51] Keogh J.W.L., Grigg J., Vertullo C.J. (2017). Is Home-Based, High-Intensity Interval Training Cycling Feasible and Safe for Patients With Knee Osteoarthritis?: Study Protocol for a Randomized Pilot Study. Orthop. J. Sports Med..

[52] Smith-Ryan A.E., Blue M.N.M., Anderson K.C., Hirsch K.R., Allen K.D., Huebner J.L., Muehlbauer M.J., Ilkayeva O.R., Kraus V.B., Kraus W.E. (2020). Metabolic and physiological effects of high intensity interval training in patients with knee osteoarthritis: A pilot and feasibility study. Osteoarthr. Cartil. Open.

[53] Verbrugghe J., Agten A., Stevens S., Hansen D., Demoulin C., Eijnde B.O., Vandenabeele F., Timmermans A. (2020). High Intensity Training to Treat Chronic Nonspecific Low Back Pain: Effectiveness of Various Exercise Modes. J. Clin. Med..

[54] Helmhout P.H., Harts C.C., Staal J.B., Candel M.J., de Bie R.A. (2004). Comparison of a high-intensity and a low-intensity lumbar extensor training program as minimal intervention treatment in low back pain: A randomized trial. Eur. Spine J..

[55] Da Cruz Fernandes I.M., Pinto R.Z., Ferreira P., Lira F.S. (2018). Low back pain, obesity, and inflammatory markers: Exercise as potential treatment. J. Exerc. Rehabil..

[56] Koes B.W., van Tulder M.W., Thomas S. (2006). Diagnosis and treatment of low back pain. BMJ.

[57] Bartlett D.B., Willis L.H., Slentz C.A., Hoselton A., Kelly L., Huebner J.L., Kraus V.B., Moss J., Muehlbauer M.J., Spielmann G. (2018). Ten weeks of high-intensity interval walk training is associated with reduced disease activity and improved innate immune function in older adults with rheumatoid arthritis: A pilot study. Arthritis Res. Ther..

[58] Norton K., Norton L., Sadgrove D. (2010). Position statement on physical activity and exercise intensity terminology. J. Sci. Med. Sport.

[59] Kjelkenes I., Thorsen E. (2010). Anticipating maximal or submaximal exercise: No differences in cardiopulmonary responses. Clin. Physiol. Funct. Imaging.

[60] Hofmann P., Tschakert G., Stark M., Schwaberger G., Pokan R., Wonisch M., Smekal G., Seibert F., Von Duvillard S. (2009). Estimation Error When Using The %HRR Method Compared To The Lactate Turn Point: 1505. Med. Sci. Sports Exerc..

[61] Wonisch M., Hofmann P., Fruhwald F.M., Kraxner W., Hödl R., Pokan R., Klein W. (2003). Influence of beta-blocker use on percentage of target heart rate exercise prescription. Eur. J. Prev. Cardiol..

[62] Midgley A.W., McNaughton L.R., Polman R., Marchant D. (2007). Criteria for determination of maximal oxygen uptake: A brief critique and recommendations for future research. Sports Med..

[63] Sabag A., Little J.P., Johnson N.A. (2021). Low-volume high-intensity interval training for cardiometabolic health. J. Physiol..

[64] Metcalfe R.S., Babraj J.A., Fawkner S.G., Vollaard N.B. (2012). Towards the minimal amount of exercise for improving metabolic health: Beneficial effects of reduced-exertion high-intensity interval training. Eur. J. Appl. Physiol..

[65] Tjønna A.E., Leinan I.M., Bartnes A.T., Jenssen B.M., Gibala M.J., Winett R.A., Wisløff U. (2013). Low- and high-volume of intensive endurance training significantly improves maximal oxygen uptake after 10-weeks of training in healthy men. PLoS ONE.

[66] Poon E.T., Little J.P., Sit C.H., Wong S.H. (2020). The effect of low-volume high-intensity interval training on cardiometabolic health and psychological responses in overweight/obese middle-aged men. J. Sports Sci..

[67] Kavaliauskas M., Steer T.P., Babraj J.A. (2017). Cardiorespiratory fitness and aerobic performance adaptations to a 4-week sprint interval training in young healthy untrained females. Sport Sci. Health.

[68] Gibala M.J., Gillen J.B., Percival M.E. (2014). Physiological and health-related adaptations to low-volume interval training: Influences of nutrition and sex. Sports Med..

[69] Gillen J.B., Gibala M.J. (2014). Is high-intensity interval training a time-efficient exercise strategy to improve health and fitness?. Appl. Physiol. Nutr. Metab..

[70] Burgomaster K.A., Hughes S.C., Heigenhauser G.J., Bradwell S.N., Gibala M.J. (2005). Six sessions of sprint interval training increases muscle oxidative potential and cycle endurance capacity in humans. J. Appl. Physiol. (1985).

[71] Gibala M.J., Little J.P., van Essen M., Wilkin G.P., Burgomaster K.A., Safdar A., Raha S., Tarnopolsky M.A. (2006). Short-term sprint interval versus traditional endurance training: Similar initial adaptations in human skeletal muscle and exercise performance. J. Physiol..

[72] Burgomaster K.A., Howarth K.R., Phillips S.M., Rakobowchuk M., Macdonald M.J., McGee S.L., Gibala M.J. (2008). Similar metabolic adaptations during exercise after low volume sprint interval and traditional endurance training in humans. J. Physiol..

[73] Richards J., Johnson T., Kuzma J., Lonac M., Schweder M., Voyles W., Bell C. (2010). Short-term sprint interval training increases insulin sensitivity in healthy adults but does not affect the thermogenic response to β-adrenergic stimulation. J. Physiol..

[74] Trapp E.G., Chisholm D.J., Freund J., Boutcher S.H. (2008). The effects of high-intensity intermittent exercise training on fat loss and fasting insulin levels of young women. Int. J. Obes..

[75] Heydari M., Freund J., Boutcher S.H. (2012). The effect of high-intensity intermittent exercise on body composition of overweight young males. J. Obes..

[76] Spencer M., Bishop D., Dawson B., Goodman C. (2005). Physiological and Metabolic Responses of Repeated-Sprint Activities. Sports Med..

[77] Bishop D., Girard O., Mendez-Villanueva A. (2011). Repeated-Sprint Ability—Part II. Sports Med..

[78] Ferrari Bravo D., Impellizzeri F.M., Rampinini E., Castagna C., Bishop D., Wisloff U. (2008). Sprint vs. interval training in football. Int. J. Sports Med..

[79] Fernandez-Fernandez J., Zimek R., Wiewelhove T., Ferrauti A. (2012). High-intensity interval training vs. repeated-sprint training in tennis. J. Strength Cond. Res..

[80] Galvin H.M., Cooke K., Sumners D.P., Mileva K.N., Bowtell J.L. (2013). Repeated sprint training in normobaric hypoxia. Br. J. Sports Med..

[81] Taylor J., Macpherson T., Spears I., Weston M. (2015). The effects of repeated-sprint training on field-based fitness measures: A meta-analysis of controlled and non-controlled trials. Sports Med..

[82] Knuttgen H.G., Nordesjö L.O., Ollander B., Saltin B. (1973). Physical conditioning through interval training with young male adults. Med. Sci. Sports.

[83] Fox E.L., Bartels R.L., Billings C.E., O’Brien R., Bason R., Mathews D.K. (1975). Frequency and duration of interval training programs and changes in aerobic power. J. Appl. Physiol..

[84] Henriksson J., Reitman J.S. (1976). Quantitative measures of enzyme activities in type I and type II muscle fibres of man after training. Acta Physiol. Scand..

[85] Roberts A.D., Billeter R., Howald H. (1982). Anaerobic muscle enzyme changes after interval training. Int. J. Sports Med..

[86] Sharp R.L., Costill D.L., Fink W.J., King D.S. (1986). Effects of eight weeks of bicycle ergometer sprint training on human muscle buffer capacity. Int. J. Sports Med..

[87] Tabata I., Nishimura K., Kouzaki M., Hirai Y., Ogita F., Miyachi M., Yamamoto K. (1996). Effects of moderate-intensity endurance and high-intensity intermittent training on anaerobic capacity and VO_2max_. Med. Sci. Sports Exerc..

[88] Meyer K., Lehmann M., Sünder G., Keul J., Weidemann H. (1990). Interval versus continuous exercise training after coronary bypass surgery: A comparison of training-induced acute reactions with respect to the effectiveness of the exercise methods. Clin. Cardiol..

[89] MacDougall J.D., Hicks A.L., MacDonald J.R., McKelvie R.S., Green H.J., Smith K.M. (1998). Muscle performance and enzymatic adaptations to sprint interval training. J. Appl. Physiol..

[90] Helgerud J., Hoydal K., Wang E., Karlsen T., Berg P., Bjerkaas M., Simonsen T., Helgesen C., Hjorth N., Bach R. (2007). Aerobic high-intensity intervals improve VO_2max_ more than moderate training. Med. Sci. Sports Exerc..

[91] Little J.P., Safdar A., Wilkin G.P., Tarnopolsky M.A., Gibala M.J. (2010). A practical model of low-volume high-intensity interval training induces mitochondrial biogenesis in human skeletal muscle: Potential mechanisms. J. Physiol..

[92] Granata C., Oliveira R.S., Little J.P., Renner K., Bishop D.J. (2016). Training intensity modulates changes in PGC-1alpha and p53 protein content and mitochondrial respiration, but not markers of mitochondrial content in human skeletal muscle. FASEB J..

[93] Granata C., Oliveira R.S., Little J.P., Renner K., Bishop D.J. (2016). Mitochondrial adaptations to high-volume exercise training are rapidly reversed after a reduction in training volume in human skeletal muscle. FASEB J..

[94] Stensvold D., Viken H., Steinshamn S.L., Dalen H., Stoylen A., Loennechen J.P., Reitlo L.S., Zisko N., Baekkerud F.H., Tari A.R. (2020). Effect of exercise training for five years on all cause mortality in older adults-the Generation 100 study: Randomised controlled trial. BMJ.

[95] Kavanagh T., Shephard R.J. (1975). Conditioning of postcoronary patients: Comparison of continuous and interval training. Arch. Phys. Med. Rehabil..

[96] Rognmo O., Hetland E., Helgerud J., Hoff J., Slordahl S.A. (2004). High intensity aerobic interval exercise is superior to moderate intensity exercise for increasing aerobic capacity in patients with coronary artery disease. Eur. J. Cardiovasc. Prev. Rehabil..

[97] Wisloff U., Stoylen A., Loennechen J.P., Bruvold M., Rognmo O., Haram P.M., Tjonna A.E., Helgerud J., Slordahl S.A., Lee S.J. (2007). Superior cardiovascular effect of aerobic interval training versus moderate continuous training in heart failure patients: A randomized study. Circulation.

[98] Rognmo O., Moholdt T., Bakken H., Hole T., Molstad P., Myhr N.E., Grimsmo J., Wisloff U. (2012). Cardiovascular risk of high- versus moderate-intensity aerobic exercise in coronary heart disease patients. Circulation.

[99] Little J.P., Gillen J.B., Percival M.E., Safdar A., Tarnopolsky M.A., Punthakee Z., Jung M.E., Gibala M.J. (2011). Low-volume high-intensity interval training reduces hyperglycemia and increases muscle mitochondrial capacity in patients with type 2 diabetes. J. Appl. Physiol..

[100] Gillen J.B., Martin B.J., MacInnis M.J., Skelly L.E., Tarnopolsky M.A., Gibala M.J. (2016). Twelve Weeks of Sprint Interval Training Improves Indices of Cardiometabolic Health Similar to Traditional Endurance Training despite a Five-Fold Lower Exercise Volume and Time Commitment. PLoS ONE.

[101] Flockhart M., Nilsson L.C., Tais S., Ekblom B., Apro W., Larsen F.J. (2021). Excessive exercise training causes mitochondrial functional impairment and decreases glucose tolerance in healthy volunteers. Cell Metab..

[102] Gibala M.J., Hawley J.A. (2017). Sprinting Toward Fitness. Cell Metab..

[103] Christensen E.H., Hedman R., Saltin B. (1960). Intermittent and Continuous Running (A further contribution to the physiology of intermittent work.). Acta Physiol. Scand..

[104] Fox E.L., Mathews D.K. (1974). Interval Training.

[105] Saleem A., Carter H.N., Iqbal S., Hood D.A. (2011). Role of p53 within the regulatory network controlling muscle mitochondrial biogenesis. Exerc. Sport Sci. Rev..

[106] Karabiyik H., Eser M.C., Guler O., Yasli B.C., Ertetik G., Sisman A., Koz M., Gabrys T., Pilis K., Karayigit R. (2021). The Effects of 15 or 30 s SIT in Normobaric Hypoxia on Aerobic, Anaerobic Performance and Critical Power. Int. J. Environ. Res. Public Health.

[107] Alarcon-Gomez J., Calatayud J., Chulvi-Medrano I., Martin-Rivera F. (2021). Effects of a HIIT Protocol on Cardiovascular Risk Factors in a Type 1 Diabetes Mellitus Population. Int. J. Environ. Res. Public Health.

[108] Iellamo F., Caminiti G., Montano M., Manzi V., Franchini A., Mancuso A., Volterrani M. (2021). Prolonged Post-Exercise Hypotension: Effects of Different Exercise Modalities and Training Statuses in Elderly Patients with Hypertension. Int. J. Environ. Res. Public Health.

[109] Jakeman J., Adamson S., Babraj J. (2012). Extremely short duration high-intensity training substantially improves endurance performance in triathletes. Appl. Physiol. Nutr. Metab..

[110] Liu H., Leng B., Li Q., Liu Y., Bao D., Cui Y. (2021). The Effect of Eight-Week Sprint Interval Training on Aerobic Performance of Elite Badminton Players. Int. J. Environ. Res. Public Health.

[111] Alansare A., Alford K., Lee S., Church T., Jung H.C. (2018). The Effects of High-Intensity Interval Training vs. Moderate-Intensity Continuous Training on Heart Rate Variability in Physically Inactive Adults. Int. J. Environ. Res. Public Health.

[112] Herget S., Reichardt S., Grimm A., Petroff D., Kapplinger J., Haase M., Markert J., Bluher S. (2016). High-Intensity Interval Training for Overweight Adolescents: Program Acceptance of a Media Supported Intervention and Changes in Body Composition. Int. J. Environ. Res. Public Health.

[113] Matsuo T., Saotome K., Seino S., Shimojo N., Matsushita A., Iemitsu M., Ohshima H., Tanaka K., Mukai C. (2014). Effects of a low-volume aerobic-type interval exercise on VO_2max_ and cardiac mass. Med. Sci. Sports Exerc..

[114] Shepherd S.O., Cocks M., Tipton K.D., Ranasinghe A.M., Barker T.A., Burniston J.G., Wagenmakers A.J., Shaw C.S. (2013). Sprint interval and traditional endurance training increase net intramuscular triglyceride breakdown and expression of perilipin 2 and 5. J. Physiol..

[115] Andrade-Souza V.A., Ghiarone T., Sansonio A., Santos Silva K.A., Tomazini F., Arcoverde L., Fyfe J., Perri E., Saner N., Kuang J. (2020). Exercise twice-a-day potentiates markers of mitochondrial biogenesis in men. FASEB J..

[116] Meyer K., Samek L., Schwaibold M., Westbrook S., Hajric R., Lehmann M., Essfeld D., Roskamm H. (1996). Physical responses to different modes of interval exercise in patients with chronic heart failure--application to exercise training. Eur. Heart J..

[117] Meyer K., Foster C., Georgakopoulos N., Hajric R., Westbrook S., Ellestad A., Tilman K., Fitzgerald D., Young H., Weinstein H. (1998). Comparison of left ventricular function during interval versus steady-state exercise training in patients with chronic congestive heart failure. Am. J. Cardiol..

[118] Bird S.R., Hawley J.A. (2016). Update on the effects of physical activity on insulin sensitivity in humans. BMJ Open Sport Exerc. Med..

[119] Trost S.G., Owen N., Bauman A.E., Sallis J.F., Brown W. (2002). Correlates of adults’ participation in physical activity: Review and update. Med. Sci. Sports Exerc..

[120] Francois M.E., Little J.P. (2015). Effectiveness and safety of high-intensity interval training in patients with type 2 diabetes. Diabetes Spectr..

[121] Burgomaster K.A., Cermak N.M., Phillips S.M., Benton C.R., Bonen A., Gibala M.J. (2007). Divergent response of metabolite transport proteins in human skeletal muscle after sprint interval training and detraining. Am. J. Physiol. Regul. Integr. Comp. Physiol..

[122] Burgomaster K.A., Heigenhauser G.J., Gibala M.J. (2006). Effect of short-term sprint interval training on human skeletal muscle carbohydrate metabolism during exercise and time-trial performance. J. Appl. Physiol..

[123] Lora-Pozo I., Lucena-Anton D., Salazar A., Galán-Mercant A., Moral-Munoz J.A. (2019). Anthropometric, Cardiopulmonary and Metabolic Benefits of the High-Intensity Interval Training Versus Moderate, Low-Intensity or Control for Type 2 Diabetes: Systematic Review and Meta-Analysis. Int. J. Environ. Res. Public Health.

[124] Liu J.X., Zhu L., Li P.J., Li N., Xu Y.B. (2019). Effectiveness of high-intensity interval training on glycemic control and cardiorespiratory fitness in patients with type 2 diabetes: A systematic review and meta-analysis. Aging Clin. Exp. Res..

[125] Qiu S., Cai X., Sun Z., Zügel M., Steinacker J.M., Schumann U. (2017). Aerobic Interval Training and Cardiometabolic Health in Patients with Type 2 Diabetes: A Meta-Analysis. Front. Physiol..

[126] Saner N.J., Lee M.J., Kuang J., Pitchford N.W., Roach G.D., Garnham A., Genders A.J., Stokes T., Schroder E.A., Huo Z. (2021). Exercise mitigates sleep-loss-induced changes in glucose tolerance, mitochondrial function, sarcoplasmic protein synthesis, and diurnal rhythms. Mol. Metab..

[127] Hawley J.A., Hargreaves M., Joyner M.J., Zierath J.R. (2014). Integrative biology of exercise. Cell.

[128] Holloszy J.O., Coyle E.F. (1984). Adaptations of skeletal muscle to endurance exercise and their metabolic consequences. J. Appl. Physiol..

[129] Mugele H., Freitag N., Wilhelmi J., Yang Y., Cheng S., Bloch W., Schumann M. (2019). High-intensity interval training in the therapy and aftercare of cancer patients: A systematic review with meta-analysis. J. Cancer Surviv..

[130] Garcia-Hermoso A., Cerrillo-Urbina A.J., Herrera-Valenzuela T., Cristi-Montero C., Saavedra J.M., Martinez-Vizcaino V. (2016). Is high-intensity interval training more effective on improving cardiometabolic risk and aerobic capacity than other forms of exercise in overweight and obese youth? A meta-analysis. Obes. Rev..

[131] Cao M., Quan M., Zhuang J. (2019). Effect of High-Intensity Interval Training versus Moderate-Intensity Continuous Training on Cardiorespiratory Fitness in Children and Adolescents: A Meta-Analysis. Int. J. Environ. Res. Public Health.

[132] Milanovic Z., Sporis G., Weston M. (2015). Effectiveness of High-Intensity Interval Training (HIT) and Continuous Endurance Training for VO_2max_ Improvements: A Systematic Review and Meta-Analysis of Controlled Trials. Sports Med..

[133] Astorino T.A., Edmunds R.M., Clark A., King L., Gallant R.A., Namm S., Fischer A., Wood K.M. (2017). High-Intensity Interval Training Increases Cardiac Output and V˙O_2max_. Med. Sci. Sports Exerc..

[134] Raleigh J.P., Giles M.D., Islam H., Nelms M., Bentley R.F., Jones J.H., Neder J.A., Boonstra K., Quadrilatero J., Simpson C.A. (2018). Contribution of central and peripheral adaptations to changes in maximal oxygen uptake following 4 weeks of sprint interval training. Appl. Physiol. Nutr. Metab..

[135] Lundby C., Montero D., Joyner M. (2017). Biology of V_O2max_: Looking under the physiology lamp. Acta Physiol..

[136] Sloth M., Sloth D., Overgaard K., Dalgas U. (2013). Effects of sprint interval training on VO_2max_ and aerobic exercise performance: A systematic review and meta-analysis. Scand. J. Med. Sci. Sports.

[137] Vollaard N.B.J., Metcalfe R.S., Williams S. (2017). Effect of Number of Sprints in an SIT Session on Change in V˙O_2max_: A Meta-analysis. Med. Sci. Sports Exerc..

[138] Bentley R.F., Jones J.H., Hirai D.M., Zelt J.T., Giles M.D., Raleigh J.P., Quadrilatero J., Gurd B.J., Neder J.A., Tschakovsky M.E. (2019). Submaximal exercise cardiac output is increased by 4 weeks of sprint interval training in young healthy males with low initial $\dot{Q}$-$\dot{V}$O2: Importance of cardiac response phenotype. PLoS ONE.

[139] Horn T., Roverud G., Sutzko K., Browne M., Parra C., Astorino T.A. (2016). Single session of sprint interval training elicits similar cardiac output but lower oxygen uptake versus ramp exercise to exhaustion in men and women. Int. J. Physiol. Pathophysiol. Pharmacol..

[140] Macpherson R.E., Hazell T.J., Olver T.D., Paterson D.H., Lemon P.W. (2011). Run sprint interval training improves aerobic performance but not maximal cardiac output. Med. Sci. Sports Exerc..

[141] Warburton D.E., Haykowsky M.J., Quinney H.A., Blackmore D., Teo K.K., Taylor D.A., McGavock J., Humen D.P. (2004). Blood volume expansion and cardiorespiratory function: Effects of training modality. Med. Sci. Sports Exerc..

[142] De Revere J.L., Clausen R.D., Astorino T.A. (2021). Changes in VO_2max_ and cardiac output in response to short-term high-intensity interval training in Caucasian and Hispanic young women: A pilot study. PLoS ONE.

[143] Astorino T.A., Edmunds R.M., Clark A., King L., Gallant R.M., Namm S., Fischer A., Wood K.A. (2018). Increased cardiac output and maximal oxygen uptake in response to ten sessions of high intensity interval training. J. Sports Med. Phys. Fitness.

[144] Krustrup P., Hellsten Y., Bangsbo J. (2004). Intense interval training enhances human skeletal muscle oxygen uptake in the initial phase of dynamic exercise at high but not at low intensities. J. Physiol..

[145] Juel C., Klarskov C., Nielsen J.J., Krustrup P., Mohr M., Bangsbo J. (2004). Effect of high-intensity intermittent training on lactate and H+ release from human skeletal muscle. Am. J. Physiol. Endocrinol. Metab..

[146] Ortenblad N., Lunde P.K., Levin K., Andersen J.L., Pedersen P.K. (2000). Enhanced sarcoplasmic reticulum Ca^2+^ release following intermittent sprint training. Am. J. Physiol. Regul. Integr. Comp. Physiol..

[147] Gabriel B.M., Zierath J.R. (2017). The Limits of Exercise Physiology: From Performance to Health. Cell Metab..

[148] Egan B., Zierath J.R. (2013). Exercise metabolism and the molecular regulation of skeletal muscle adaptation. Cell Metab..

[149] Li J., Li Y., Atakan M.M., Kuang J., Hu Y., Bishop D.J., Yan X. (2020). The Molecular Adaptive Responses of Skeletal Muscle to High-Intensity Exercise/Training and Hypoxia. Antioxidants.

[150] Egan B., Carson B.P., Garcia-Roves P.M., Chibalin A.V., Sarsfield F.M., Barron N., McCaffrey N., Moyna N.M., Zierath J.R., O’Gorman D.J. (2010). Exercise intensity-dependent regulation of peroxisome proliferator-activated receptor coactivator-1 mRNA abundance is associated with differential activation of upstream signalling kinases in human skeletal muscle. J. Physiol..

[151] Di Donato D.M., West D.W., Churchward-Venne T.A., Breen L., Baker S.K., Phillips S.M. (2014). Influence of aerobic exercise intensity on myofibrillar and mitochondrial protein synthesis in young men during early and late postexercise recovery. Am. J. Physiol. Endocrinol. Metab..

[152] Bishop D.J., Granata C., Eynon N. (2014). Can we optimise the exercise training prescription to maximise improvements in mitochondria function and content?. Biochim. Biophys. Acta Gen. Subj..

[153] Granata C., Jamnick N.A., Bishop D.J. (2018). Training-Induced Changes in Mitochondrial Content and Respiratory Function in Human Skeletal Muscle. Sports Med..

[154] Philp A.M., Saner N.J., Lazarou M., Ganley I.G., Philp A. (2020). The influence of aerobic exercise on mitochondrial quality control in skeletal muscle. J. Physiol..

[155] Combes A., Dekerle J., Webborn N., Watt P., Bougault V., Daussin F.N. (2015). Exercise-induced metabolic fluctuations influence AMPK, p38-MAPK and CaMKII phosphorylation in human skeletal muscle. Physiol. Rep..

[156] Ijichi T., Hasegawa Y., Morishima T., Kurihara T., Hamaoka T., Goto K. (2015). Effect of sprint training: Training once daily versus twice every second day. Eur. J. Sport Sci..

[157] Tsuchiya Y., Ijichi T., Goto K. (2016). Effect of sprint training on resting serum irisin concentration - Sprint training once daily vs. twice every other day. Metabolism.

[158] Ghiarone T., Andrade-Souza V.A., Learsi S.K., Tomazini F., Ataide-Silva T., Sansonio A., Fernandes M.P., Saraiva K.L., Figueiredo R., Tourneur Y. (2019). Twice-a-day training improves mitochondrial efficiency, but not mitochondrial biogenesis, compared with once-daily training. J. Appl. Physiol..

[159] Hammond K.M., Sale C., Fraser W., Tang J., Shepherd S.O., Strauss J.A., Close G.L., Cocks M., Louis J., Pugh J. (2019). Post-exercise carbohydrate and energy availability induce independent effects on skeletal muscle cell signalling and bone turnover: Implications for training adaptation. J. Physiol..

[160] Cochran A.J., Myslik F., MacInnis M.J., Percival M.E., Bishop D., Tarnopolsky M.A., Gibala M.J. (2015). Manipulating Carbohydrate Availability Between Twice-Daily Sessions of High-Intensity Interval Training Over 2 Weeks Improves Time-Trial Performance. Int. J. Sport Nutr. Exerc. Metab..

[161] Yeo W.K., Paton C.D., Garnham A.P., Burke L.M., Carey A.L., Hawley J.A. (2008). Skeletal muscle adaptation and performance responses to once a day versus twice every second day endurance training regimens. J. Appl. Physiol..

[162] Cochran A.J., Little J.P., Tarnopolsky M.A., Gibala M.J. (2010). Carbohydrate feeding during recovery alters the skeletal muscle metabolic response to repeated sessions of high-intensity interval exercise in humans. J. Appl. Physiol..

[163] Hulston C.J., Venables M.C., Mann C.H., Martin C., Philp A., Baar K., Jeukendrup A.E. (2010). Training with low muscle glycogen enhances fat metabolism in well-trained cyclists. Med. Sci. Sports Exerc..

[164] Gejl K.D., Vissing K., Hansen M., Thams L., Rokkedal-Lausch T., Plomgaard P., Meinild Lundby A.K., Nybo L., Jensen K., Holmberg H.C. (2018). Changes in metabolism but not myocellular signaling by training with CHO-restriction in endurance athletes. Physiol. Rep..

[165] Psilander N., Frank P., Flockhart M., Sahlin K. (2013). Exercise with low glycogen increases PGC-1alpha gene expression in human skeletal muscle. Eur. J. Appl. Physiol..

[166] Hansen A.K., Fischer C.P., Plomgaard P., Andersen J.L., Saltin B., Pedersen B.K. (2005). Skeletal muscle adaptation: Training twice every second day vs. training once daily. J. Appl. Physiol..

[167] Fletcher G.F., Balady G.J., Amsterdam E.A., Chaitman B., Eckel R., Fleg J., Froelicher V.F., Leon A.S., Piña I.L., Rodney R. (2001). Exercise standards for testing and training: A statement for healthcare professionals from the American Heart Association. Circulation.

[168] Mittleman M.A., Maclure M., Tofler G.H., Sherwood J.B., Goldberg R.J., Muller J.E. (1993). Triggering of Acute Myocardial Infarction by Heavy Physical Exertion -- Protection against Triggering by Regular Exertion. N. Engl. J. Med..

